# Saline pasture improve meat quality in Qinghai Tibetan sheep through changes in the rumen microbiota

**DOI:** 10.3389/fmicb.2025.1573040

**Published:** 2025-08-05

**Authors:** Yujiao Jia, Lijuan Han, Shengzhen Hou, Linsheng Gui, Zhenzhen Yuan, Shengnan Sun, Zhiyou Wang, Baochun Yang, Chao Yang

**Affiliations:** College of Agriculture and Animal Husbandry, Qinghai University, Xining, China

**Keywords:** Tibetan sheep, saline-alkali stress, forage quality, meat quality, metabolomics, rumen microorganism

## Abstract

**Introduction:**

This study aimed to investigate the effects of natural forage from different regions (saline-alkali and non-saline-alkali areas) on the rumen microbiota, muscle metabolites, and meat quality of Tibetan sheep.

**Methods:**

Targeted and non-targeted metabolomics were used to comprehensively analyze both pasture and meat quality, metabolites. Additionally, 16S rDNA sequencing was employed to analyze the rumen microbial community structure of Tibetan sheep.

**Results:**

The results showed that the natural saline-alkali forage (HG group) had higher protein content, lower fiber content, higher relative feed value, and better quality. Metabolomic analysis revealed significant accumulation of flavonoids and upregulation of amino acid metabolism in the HG group. Additionally, the natural saline-alkali forage significantly increased amino acid deposition in Tibetan sheep muscle, markedly enhanced the redness value (a*), and significantly reduced the yellowness value (b*). Furthermore, the natural saline-alkali forage altered the rumen fermentation patterns in Tibetan sheep, leading to a significant increase in the abundance of F082 and WCHB1-41, while significantly reducing the abundance of Prevotellaceae_UCG-003. Correlation analysis revealed that these microbial taxa were significantly influenced by the natural saline-alkali forage,while also showing significant associations with muscle quality parameters (a*, b*) and metabolites (cysteine, C18:1n9, etc.).

**Discussion:**

Overall, the natural saline-alkali forage demonstrated superior quality and metabolite content compared to natural non-saline-alkali forage. Furthermore, this saline-alkali forage significantly influenced the abundance of specific rumen microbiota in Tibetan sheep, consequently regulating muscle coloration and amino acid deposition.

## Graphical abstract

The quality and metabolism of saline pasture and meat quality, metabolism and rumen fermentation of Tibetan sheep were studied and correlation analysis was done to elucidate the possible mechanisms by which saline pasture affects meat quality of Tibetan sheep. 
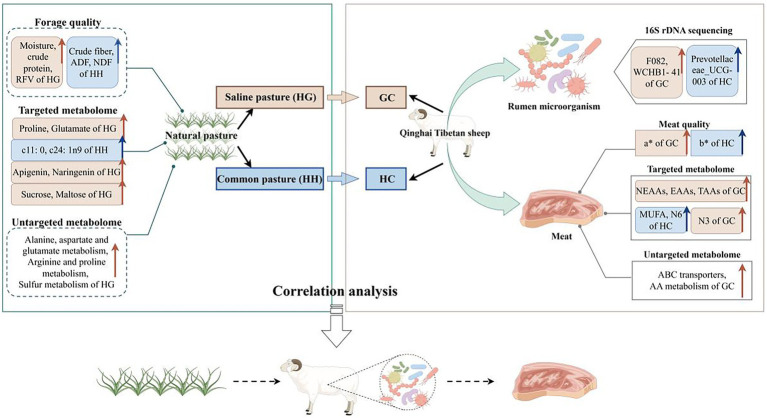

## 1 Introduction

Saline soils are characteristically highly alkaline with high salt levels. These conditions cause severe stress to plants, adversely affecting growth. Saline regions thus tend to be sparsely vegetated and ecologically fragile (Wang W. et al., 2024; Wang H. et al., 2024). Certain forage plants, such as *Achnatherum splendens, Puccinellia distans* (L.) Parl., *Nitraria tangutorum* Bobr., and *Salsola ruthenica* Iljin, are well-adapted to high salinity and can tolerate and grow normally in regions with saline and alkaline soils (Zhang, 2008). Previous studies have shown that in response to saline stress, plants accumulate a variety of beneficial substances, enhancing the quality of the plant. For instance, Cui et al. (2025) observed that salt-tolerant *Cynodon dactylon* exhibited enhanced crude protein accumulation capability under salt stress, with the crude protein content in the leaves of the ‘Wrangler’ cultivar of *Cynodon dactylon* increasing by 44.20% compared to the control group. This characteristic suggests that salt-tolerant plants possess higher nutritional value as animal feed. Lv et al. (2024) reported that the leaves of the salinity-tolerant plant *Glycyrrhiza uralensis* are rich in flavonoids, a type of antioxidant substance, which act as non-enzymatic antioxidants in response to saline stress. Sun et al. (2019) found that saline stress stimulated the effective accumulation of two organic osmolytes, soluble proteins and proline, in *Sorghum bicolor* seedlings, resulting in increased resistance to saline stress through the maintenance of the intracellular water potential and the regulation of osmotic pressure. Furthermore, Jiang et al. (2022) research demonstrated that under salt stress, the content of neutral detergent fiber (NDF) and acid detergent fiber (ADF) in *Paspalum vaginatum* O. *Swartz* decreased, while the relative feed value (RFV) increased, indicating superior forage quality.

Livestock living in saline areas tend to produce better-quality meat. For example, the meat of the Ningxia tan sheep, compared with other sheep breeds, has both a higher fat content and increased concentrations of fatty acids and amino acids (Zhang, 2018; Li et al., 2023; Zhang et al., 2023; Zhang et al., 2023). The high quality of meat from Chaka sheep grazing in high-salt environments was found to result from increased biosynthesis of organic and fatty acids in the longissimus dorsi muscles of the back (Guo T. et al., 2022). These observations have led to the suggestion that the type of pasture influences the quality of livestock meat. Qiu et al. (2023) the effects of alfalfa grown under saline conditions on the growth, development, and meat quality of meat lambs, finding that the average daily intake of lambs fed on alfalfa from saline soils was significantly increased, while the drip loss and cooking loss were significantly reduced, although no marked effects were observed in indices such as average daily weight gain, slaughter performance, pH, and shear energy did not have a significant difference, indicating that saline pasture had a beneficial effect on the growth and development of the lambs and their subsequent meat quality.

In Qinghai Province, China, saline and alkaline land covers an area of 3.2 million hectares (48 million mu), of which 2% is distributed in the Chaka and Gonghe basins as well as in the eastern agricultural areas. However, there have been few studies on the quality of pasture grasses growing in saline and alkaline land in Gonghe County, with no in-depth investigations of pasture quality. The Tibetan sheep living in this area consume this salt-tolerant pasture, and their meat is nutrient-rich, with excellent taste and low odor (Zhang et al., 2022b). Moreover, with the increasing public awareness of healthy living, Tibetan mutton, which is high in protein, low in fat, rich in minerals, and high in unsaturated fatty acids, is considered to have the characteristics of “green food”(Chen et al., 2018; Jiao et al., 2020; Zhongla et al., 2024). Therefore, it is gaining more and more attention and recognition from consumers. However, the ways in which consumption of salt-tolerant pasture influences the quality of Tibetan lamb meat are not clear.

Ruminal microorganisms are known to play important roles in the digestion of ruminants, and fluctuations in their abundance and community compositions can reflect changes in both the feed and rumen function, which in turn can influence the quality of the meat (Ma et al., 2023b; Guo et al., 2024b). For example, Guo W. et al. (2022) found that the effect of diet on rumen microbial communities was dramatic. Zheng et al. (2025) reported that dietary supplementation with 20% *Broussonetia papyrifera* silage enhanced the abundance of beneficial bacterial populations in the rumen of Kazakh lamb. Furthermore, Compared to a dietary combination of corn stover and salt-tolerant pasture, lambs consuming an exclusive diet of salt-tolerant pasture had significantly higher rumen pH, while digestibility of neutral detergent fiber (NDF) and crude protein (CP) was found to be markedly reduced by the addition of salt-tolerant pasture (Wang C. et al., 2011). Here, we hypothesized that Tibetan sheep grazing in areas where the quality of the pasture differs significantly would show differences in the composition of the rumen microbiota and thus in the quality of the meat.

Currently, the mechanistic effects of natural saline-alkali forage on the meat quality of Tibetan sheep remain unclear. Furthermore, the alterations in rumen microbiota induced by natural saline-alkali forage and their relationship with Tibetan sheep meat quality have not yet been elucidated. Therefore, this study analyed the quality and metabolite contents of pasture and meat using ultra-high performance liquid chromatography with quadrupole time-of-flight mass spectrometry (UHPLC-Q-TOF MS) and determined the composition of the rumen microbiota through 16S rDNA sequencing. The aim was to explore the relationships between forage quality and metabolites, key bacteria in the rumen of Tibetan sheep, and the quality and metabolites of Tibetan sheep meat, providing new research ideas for the in-depth analysis of the mechanisms responsible for the quality of Tibetan sheep meat in saline and alkaline areas.

## 2 Materials and methods

The study was carried out at the Tibetan Sheep Breeding Center in Gonghe County, Qinghai Province approved by the Animal Ethics Committee of Qinghai University (NO. QUA-2022-1972, 2022.07.02).

### 2.1 Collection of forage samples

Samples of natural mixed forage grasses were collected from the saline and alkaline area of Gonghe County, Qinghai Province (latitude: 36°28′2”N; longitude: 99°16′26″E; altitude: 3168.1 m) on August 30, 2023, and were recorded as HG. A total of 28 species of mixed forage were collected, with specific names listed in Table 1. Natural mixed forage grasses (21 species) were also collected from the non-saline area of Haiyan County, Qinghai Province (latitude: 36°59′36”N; longitude: 100°55′53″E; altitude: 3111.0 m) on August 29, 2023, and recorded as HH, with specific names presented in Table 1. For pasture collection, six sample squares were randomly selected from the natural pasture using 0.5 × 0.5 m sample frames, with the pasture mown 2–3 cm above the ground in the sample squares, placed in sampling bags, transferred to the laboratory, and stored at −80°C for further analysis.

**Table 1:** The types and names of natural mixed forages in different regions.

	Types and names of mixed forage grasses
Salt-Alkali Tolerant Grass Mixtures	*Stipa breviflora*, *Sonchus arvensis*, *Hedysarum multijugum, Limonium aureum, Suaeda corniculata, Salsola kali, Heteropappus altaicus*, *Distichlis spicata*, *Thermopsis lanceolata, Artemisia scoparia, Reaumuria soongarica, Reaumuria soongorica, Agropyron cristatum, Artemisia arenaria, Caragana tibetica, Asterothamnus centrali-asiaticus, Bassia dasyphylla, Ceratoides latens, Salsola arbuscula, Artemisia pectinata, Atriplex sibirica, Euphorbia scorpioides, Artemisia frigida, Puccinellia distans, Achnatherum splendens, Leymus secalinus*, *Nitraria tangutorum, Allium tanguticum*
Non-Saline-Alkali Grass Mixtures	*Plantago asiatica*, *Thermopsis lanceolata, Astragalus scaberrimus, Ajania tenuifolia, Gentiana scabra, Gentianopsis barbata, Artemisia argyi, Elymus dahuricus* var. *Violaceus, Potentilla bifurca, Kobresia humilis, Pedicularis, Gentiana macrophylla, Potentilla anserina, Bupleurum chinense, Gentiana dahurica, Sarcocornia, Heteropappus altaicus*, *Comastoma, Stipa krylovii, Elymus nutans, Poa*

### 2.2 Experimental animals and husbandry management

Sixty healthy Tibetan rams aged 2–3 months with an initial weight of 13.42 ± 0.62 kg were selected and randomly divided into two groups, namely, the saline (Gonghe) grazing group (GC, *n* = 30) and the non-saline (Haiyan) grazing group (HC, *n* = 30). A group feeding approach was used, with the Tibetan sheep in the GC group grazing on the desert grassland in Gonghe County, Qinghai Province (36°28′2”N, 99°16′26″E, altitude: 3168.1 m), where they were free to feed on the local natural saline pasture grasses (mainly including *Achnatherum splendens, Ceratoides lateens, Puccinellia distans* (L.) Parl., *Bassia dasyphylla* O. kuntze, *Nitraria tangutorum* Bobr., and *Salsola ruthenica* Iljin) from 08:00 to 18:00. In the HC group, the Tibetan sheep were kept in the grazing area of Haiyan County, Qinghai Province (36°59′36”N, 100°55′53″E, altitude: 3111.0 m), where they fed freely on local common forage grasses (mainly including *Gentiana, Poa, Stipa krylovii, Elymus purpuraristatus* C. P, and *Elymus nutans*) from 08:00 to 18:00. All animals had free access to water, and all animals were uniformly vaccinated and dewormed, and had undergone a 7-day acclimatisation period before being allowed to graze, followed by 150 days of formal experimentation. In addition, the sheep housing was swept and the water troughs were cleaned daily and sterilized weekly.

At the end of the trial, six test animals were randomly selected from each group, and after 12 h of fasting (both solid and liquid fasting according to animal welfare procedures), the animals were transported to the humane slaughter facility on October 11, 2023 (the distance from the pasture to the slaughterhouse was essentially the same for all Tibetan sheep). In accordance with animal welfare procedures, all animals were humanely slaughtered: firstly, the animals were electrocuted to induce unconsciousness, causing it to lose pain sensation, and then bleed it to death. The *longissimus dorsi* muscles were sampled from both sides of the carcass between the 9th and 11th ribs for subsequent analyses, and slaughtering and sampling were carried out simultaneously by professionals according to standardized norms. Samples were stored in dry ice immediately after sampling and all samples were transferred to the laboratory for storage at −80°C for further analysis. Six biological replicates and three technical replicates were obtained in each group for assessment of carcass and meat quality, amino acid (AA) and fatty acid (FA) composition, muscle metabolomics, and rumen microbiota studies.

### 2.3 Analysis of forage quality

#### 2.3.1 Nutrient analysis of forage grasses

The forage samples were heated and dried in an oven (DHG-9070A, Shanghai Yiheng Scientific Instrument Co., Ltd., Jiangsu, China) at 105 ± 1°C until constant weight for measurement of the moisture content. The Kjeldahl method was used to determine the crude protein, and the Soxhlet extraction method was used to measure the crude fat content in the pasture. The crude fiber content was measured using acid–base fractionated hydrolysis (Zhang et al., 2024). Neutral detergent fiber (NDF) and acid detergent fiber (ADF) contents were determined using the VanSoest detergent fiber assay, as described by Zhang L. et al. (2023). Ultrasound-assisted extraction of flavonoids was carried out using Deng et al. (2024) method, and the final crude polysaccharides were determined using the phenol sulfate method (Tepsongkroh et al., 2023).

#### 2.3.2 Calculation of quality indices of forages

The method of Gao et al. (2023) was used to calculate the relative feeding value (RFV) of the pasture using the formula:


RFV=(DMI(%DW)×DDM(%DW))/1.29

$$\begin{array}{l} \mathrm{DMI}=120/\mathrm{NDF}\\ \mathrm{DDM}=88.9-(0.779\times \mathrm{ADF}) \end{array}$$

where DMI represents the dry matter intake and DDM indicates the digestible dry matter. Higher RFV values represent better quality of forage.

### 2.4 Quality analysis of Tibetan sheep

#### 2.4.1 Carcass quality analysis

Tibetan sheep were weighed during carcass splitting to obtain raw data on their carcass quality. The eye muscle area (EMA) was obtained by tracing the cross-section of the EMA with sulfuric acid paper at the time of slaughter and then calculating the area. Rib fat thickness (RFT), abdominal fat thickness (AFT) and back fat thickness (BFT) were then measured directly with vernier calipers at 110 mm, 127 mm, and 40 mm, respectively, from the spine (Zhang et al., 2022a).

#### 2.4.2 Sensory quality analysis of the longissimus dorsi muscle

Sensory evaluation of the Tibetan sheep samples was performed as described by Ma et al. (2023b). We invited 10 professionals (5 males and 5 females) aged between 21 and 25 to evaluate the meat samples. Prior to evaluation, all panelists completed specialized sensory training and demonstrated proficiency in meat product assessment protocols. The two groups of cooked meat samples were portioned into 10 aliquots, each labeled with random 3-digit codes. During assessment, panelists slowly masticated the samples and rated them according to six sensory attributes (color, fragrance, muttony odor, taste, texture, and general acceptability) using the predefined scoring criteria (Supplementary Table S1). The judges were seated in a controlled room, with each individual occupying a separate cubicle, and the environmental conditions were kept as consistent as possible. During the evaluation, each judge scored two sets of samples received at random. A few hours were spaced between the two evaluations, during which the judges were provided with pure water to thoroughly rinse their mouths. Communication among the judges was not permitted. The results were calculated by averaging the scores provided by the judges.

#### 2.4.3 Analysis of the edible quality of the longissimus dorsi muscle

The edible quality was assessed with reference to meat quality testing procedures. The probe of a pH meter (pHS-S3C, Shanghai Yoke Instrument Co., Ltd., Shanghai, China) calibrated to pH 4.0 and 6.86 was inserted into the center of the meat sample to measure the pH. The chromaticity values (a* [red], b* [yellow], and L* [lightness]) of the *longissimus dorsi* muscle were then determined using an auto-calibrated colorimeter (ADCI, Beijing Chentaike Instrument Technology Co., Ltd., Beijing, China). Thawing loss was measured by thawing the samples at 4°C for 12 h, while cooking loss and the cooking rate of the meat samples were measured by steaming the samples at 80°C for 30 min and 40 min, respectively, in a thermostatic water bath (HH-6, Changzhou Jintan Youlian Instrument Research Institute, Jiangsu, China). In addition, the thawed samples were divided into 2 × 1 × 1 cm pieces along the direction of the muscle fiber, and the shear force was determined using a tenderness meter (MAQC-12, Nanjing Xiyi Instrument Equipment Co., Ltd., China). Finally, the hardness, elasticity, stickiness, adhesion, and chewability of the meat samples were assessed using a texturometer (CT3-10 K, Brookfield Engineering Laboratories, Inc., USA).

### 2.5 Targeted metabolomics analysis

#### 2.5.1 Analysis of amino acid composition

Sixty milligrams of sample were mixed with 50 μL of water and homogenized and vortexed for 60 s. This was followed by the addition of 400 μL of methanol acetonitrile solution and 50 μL of internal standard mix (50 μm of 16 isotopes), vortexed for 60 s, and sonicated at low temperature for 30 min (repeated twice), after which the mixture was left for 1 h at −20°C to precipitate the proteins, before centrifugation at 14000 rpm for 20 min at 4°C and the supernatant was discarded. The samples were freeze-dried and stored at −80°C. The samples were then separated using an Agilent 1,290 Infinity UHPLC system (Agilent Technologies, USA) and analyzed by mass spectrometry using a 6500/5500 QTRAP mass spectrometer (SCIEX) in positive ion mode.

#### 2.5.2 Analysis of fatty acid composition

The samples were thawed at 4°C and 5 mL of dichloromethane-methanol solution (2:1 v/v) was added, followed by vortexing and the addition of 2 mL of gold standard water to wash and collection of the lower layer for drying under nitrogen. Two milliliters of n-hexane were added and methylated for 0.5 h, followed by the addition of 2 mL of gold standard water, after which 2000 μL of the supernatant was collected, dried under nitrogen and re-dissolved n-hexane. The supernatant was placed in an injection vial and analyzed by gas chromatography using a capillary column (Agilent 19091S-433UI: HP-5 ms, 30 mx 250 μm x 0.25 μm) GC system to separate the samples. Mass spectrometry analysis was then performed using a 5977B MSD mass spectrometer (Agilent).

#### 2.5.3 Analysis of flavonoid composition of pasture grasses

One hundred milligrams of samples stored at −80°C were added to 300 μL of extraction solution and 10 μL of internal standard (10 μg/mL), followed by thorough vortexing for 30 s, sonication for 0.5 h in a water bath, centrifugation at 14,000 g for 20 min at 10°C, and aspiration of the supernatant into an Ostro 25 mg 96-well plate (Waters, 186,005,518). Filtration was performed using a positive-pressure device, and 200 μL of the extraction solution was added to the wells for elution. Separation was performed on an Agilent 1,290 Infinity LC ultra-high performance liquid chromatography (UHPLC) system. A 5500 QTRAP mass spectrometer (SCIEX) was used for mass spectrometry in the positive/negative ion mode.

#### 2.5.4 Analysis of monosaccharide composition of pasture grasses

The samples were freeze-dried under vacuum and then ground to powder (30 Hz, 1.5 min) with a ball mill, after which 20 mg was weighed into the corresponding numbered centrifuge tube, 500 μL of methanol: isopropanol: water (3:3:2, v/v/v) was added, followed by vortexing for 3 min, sonication in a water bath at 4°C for 30 min, and centrifugation at 1200 rpm for 3 min at 4°C. Fifty microliters of the supernatant were aspirated, and 20 μL of internal standard solution with a concentration of 1,000 μg/mL was added to the sample. The solution was dried under nitrogen and lyophilized. One hundred microliters of pyridine methoxide ammonium salt (15 mg/mL) were then added and incubated at 37°C for 2 h. This was followed by the addition of 100 μL of BSTFA and incubation at 37°C for 30 min to obtain the derivatized solution, of which 50 μL was removed and diluted to 1 mL by adding n-hexane. The solution was filtered through a 0.22 μm membrane, and the filtrate was preserved in a brown injection flask and used for GC–MS (8890-5977B, Agilent) analysis. The chromatographic and mass spectrometric acquisition conditions were as follows: injection volume 1 μL; split mode 5:1; column DB-5MS (30 m x 0.25 mm x 0.25 μm); column flow rate 1 mL/min; ion source temperature 230°C; quadrupole temperature 150°C; ionization voltage 70 eV.

### 2.6 Non-targeted metabolomics analysis

The samples were thawed slowly at 4°C, and the appropriate amount was added into pre-cooled methanol/acetonitrile/water solution (2:2:1, v/v), vortexed and mixed, sonicated for 30 min at low temperature, allowed to stand at −20°C for 10 min, and then centrifuged at 14,000 g at 4°C for 20 min, after which the supernatant was retained and vacuum-dried. The samples were analyzed by UHPLC-QTOF-MS(1,290 Infinity LC, Agilent) with an AB Triple TOF 6600 mass spectrometer, and the separation columns were selected from HILIC and ESI sources using the conditions previously described (Ma et al., 2023a).

### 2.7 Quantification and analysis of bacterial diversity in the rumen microbiota

The rumen fluid removed from the Tibetan sheep at slaughter was filtered into 50 mL centrifuge tubes for the determination of pH, and the short-chain fatty acids were analyzed by gas chromatography.

Rumen fluid samples stored at −80°C were thawed under running water, and the genomic DNA of the samples was extracted using the OMEGA Mag-bind soil DNA kit, followed by amplification and sequencing of the V3-V4 variable region on an Illumina NovaSeq 6,000 sequencing platform, as described by Zhang et al. (2022b). PCR products were analyzed by 2% agarose gel electrophoresis, and the target fragments were cut and recovered by gel recovery using Quant-iT PicoGreen dsDNA Assay Kit, quantification of the products using an FLx800 microplate reader (BioTek, USA) fluorescence quantification system. Library construction was then performed using the TruSeq Nano DNA LT Library Prep Kit. Prior to sequencing, the quality of the library was assessed using an Agilent Bioanalyzer 2,100 and Promega QuantiFluor. After sequencing, the primer sequences were removed using cutadapt software, and then the quality of the paired-end raw reads was assessed using the QIIME 2 default parameters using DADA2. Representative sequences and ASV abundance tables were obtained. The representative sequences were annotated and classified against the Silva (version 138) database using the QIIME 2 software package. The results were processed and analyzed.

### 2.8 Data processing and analysis

Differences between indicators of Tibetan sheep carcass, edible, and nutritional quality between saline and non-saline areas were analyzed using independent samples *t*-tests in SPSS version 26.0 (IBM Corp., Armonk, NY, USA), with *p* < 0.05 considered statistically significant. In addition, the nutritional composition (crude protein, crude fat, and moisture content) of *longissimus dorsi* muscle was determined following standard AOAC procedures (Al-Mentafji, 2016). Correlations between forage quality and metabolism, meat quality and meat metabolism, and forage metabolism and Tibetan sheep metabolism were analyzed using Pearson’s correlation coefficients (*p* < 0.05 indicates a significant correlation, and *p* < 0.01 indicates an extremely significant correlation). Furthermore, PLS-DA and OPLS-DA were performed using SIMCA software (version 14.1). For metabolomics data analysis, the XCMS software platform was employed for metabolite identification and quantification, including peak alignment, retention time correction, and peak area extraction. Specifically, functional annotation of differential metabolites and their associated metabolic pathway analysis were performed using the Kyoto Encyclopedia of Genes and Genomes (KEGG) database (KEGG, www.genome.jp/kegg). *α*, *β* diversity indices of rumen samples were calculated using QIIME 2.

## 3 Results

### 3.1 Analysis of forage quality and metabolites

#### 3.1.1 Nutrient composition of forages

Differences in the quality of natural pasture in different regions are shown in Table 2. Significant differences were found in nutrient levels between the HG and HH groups, with the HG saline pasture group having significantly higher moisture, crude protein, and crude fat contents than the HH non-saline pasture group (*p <* 0.05), while the contents of crude fiber, NDF, and ADF were significantly lower in the HH group (*p <* 0.05).

**Table 2:** Analysis of the quality of natural forages from different regions.

Parameters	HG	HH	*p*-value
Moisture (%)	64.14 ± 3.08**	53.35 ± 3.08	0.01
Crude protein (%)	9.12 ± 0.02*	8.59 ± 0.21	0.05
Crude fat (%)	3.22 ± 0.12	3.05 ± 0.32	0.47
Flavonoid (mg/100 g)	1.59 ± 0.06	1.56 ± 0.04	0.43
Polyphenol(mg/100 g)	18.72 ± 0.00	18.56 ± 0.30	0.46
Crude fibers (%)	10.70 ± 0.20	14.07 ± 0.40**	<0.01
NDF (%)	71.63 ± 0.60	75.63 ± 0.96**	0.01
ADF (%)	41.57 ± 2.03	47.50 ± 1.22*	0.02
DDM%	56.52 ± 1.58*	51.90 ± 0.95	0.02
DMI%	1.68 ± 0.01**	1.59 ± 0.02	0.01
RFV%	73.41 ± 2.57**	63.83 ± 1.25	0.01

Values (mean ± SD). *p< 0.05 and **p < 0.01. HH: natural saline-alkali mixed forages; HG: natural common mixed forages; DDM: digestible dry matter; DMI: dry matter intake; RFV: relative feeding value.

#### 3.1.2 Calculation of forage quality indicators

As shown in Table 2, the DDM, DMI, and RFV values were greater in the HG group compared to the HH group (*p <* 0.05), indicating that the HG saline pasture was better in quality with greater nutritional value for ruminants.

#### 3.1.3 Targeted metabolomics analysis of forage grasses

##### 3.1.3.1 Analysis of the amino acid composition of forage grasses

Differences in the composition and contents of amino acids in natural pasture grasses from different regions are shown in Table 3. As can be seen from the table, the amino acid contents differed significantly between the HG and HH groups. Specifically, the HG group has a significantly higher level of aminoadipic acid, arginine, citrulline, creatinine, cysteine, cystine, glutamate, glycine, histidine, hydroxyproline, proline, and valine than in the HH group, whereas the contents of alanine, asparagine, choline, creatine, glutamine, isoleucine, ornithine, phenylalanine, serine, taurine, and threonine were higher in the HH group (*p <* 0.05).

**Table 3:** Analysis of amino acid composition and content in natural forages from different regions (mg/100 g).

Parameters	HG	HH	*p*-value
Alanine	177.60 ± 4.24	269.68 ± 1.61**	<0.01
Aminoadipic acid	15.59 ± 1.53**	4.17 ± 0.15	0.01
Arginine	1678.43 ± 92.45**	710.93 ± 234.20	0.01
Asparagine	16.62 ± 0.05	22.53 ± 0.61**	<0.01
Choline	217.28 ± 1.65	254.43 ± 4.07**	<0.01
Citrulline	1.96 ± 0.14**	0.86 ± 0.07	<0.01
Creatine	0.37 ± 0.01	0.62 ± 0.01**	<0.01
Creatinine	0.05 ± 0.00**	0.01 ± 0.00	<0.01
Cysteine	0.07 ± 0.01*	0.06 ± 0.00	0.04
Cystine	0.84 ± 0.00**	0.44 ± 0.00	<0.01
Glutamate	811.07 ± 4.52**	668.89 ± 6.32	<0.01
Glutamine	0.02 ± 0.01	0.07 ± 0.01**	<0.01
Glycine	509.08 ± 59.50*	275.31 ± 8.66	0.02
Histidine	2194.89 ± 90.68**	963.16 ± 266.49	0.01
Hydroxyproline	1.45 ± 0.00**	0.55 ± 0.02	<0.01
Isoleucine	361.49 ± 6.88	425.51 ± 5.33**	<0.01
Ornithine	0.27 ± 0.02	0.46 ± 0.02**	<0.01
Phenylalanine	965.98 ± 38.67	1077.94 ± 0.77*	0.04
Proline	4740.09 ± 228.26**	554.81 ± 10.61	<0.01
Serine	1202.01 ± 19.60	1406.31 ± 7.02**	<0.01
Taurine	0.04 ± 0.01	0.42 ± 0.05**	<0.01
Threonine	1637.87 ± 18.88	3171.25 ± 27.81**	<0.01
Valine	1531.05 ± 29.34**	1025.76 ± 3.41	<0.01
EAAs	4861.93 ± 22.14	6107.38 ± 19.95**	<0.01
NEAAs	11912.38 ± 430.30**	5479.39 ± 485.33	<0.01
TAAs	16774.31 ± 452.44**	11586.76 ± 465.38	<0.01

Values (mean ± SD). *p < 0.05 and **p < 0.01.

##### 3.1.3.2 Analysis of the fatty acid composition of forage grasses

There was a significant difference in the fatty acid content between the HG and HH groups (Table 4). In this case, Compared with the HH group, the contents of fatty acids such as C10: 0, C18: 1N9, and C18: 2N6 were significantly higher in the HG group (*p* < 0.05). In contrast, the contents of fatty acids such as C11:0, C16:1N7, and C18:3N3 were significantly higher in the HH group (*p* < 0.05). Moreover, C18:3N3 is the most abundant fatty acid in natural forage feed. Overall, mono-unsaturated fatty acids (MUFAs), and N6 polyunsaturated fatty acids (PUFAs) levels, as well as the N6/N3 ratio were considerably higher in the HG group, while the content of N3 PUFAs was significantly higher in the HH group (*p <* 0.05).

**Table 4:** Analysis of fatty acid composition and content in natural forages from different regions (mg/100 g).

parameters	HG	HH	*p*-value
c10:0	0.35 ± 0.09*	0.12 ± 0.01	0.05
c11:0	0.01 ± 0.00	0.02 ± 0.00**	0.01
c15:0	0.44 ± 0.01	0.46 ± 0.00*	0.02
c16:0	49.81 ± 0.27	52.57 ± 0.63**	0.01
c16:1n7	0.71 ± 0.01	1.02 ± 0.01**	<0.01
c17:1n7	0.19 ± 0.04	0.38 ± 0.03**	<0.01
c18:0	10.78 ± 0.05**	8.79 ± 0.20	<0.01
c18:1n9	36.53 ± 2.46**	13.30 ± 0.45	<0.01
c18:2n6	82.88 ± 3.61**	53.30 ± 0.25	0.01
c18:3n6	0.01 ± 0.00	0.03 ± 0.00**	0.01
c18:3n3	110.57 ± 2.39	141.96 ± 0.74**	<0.01
c20:1n9	2.22 ± 0.09	2.48 ± 0.08*	0.02
c20:2n6	0.22 ± 0.00**	0.20 ± 0.00	<0.01
c20:3n3	0.24 ± 0.03	0.39 ± 0.02**	<0.01
c22:0	0.04 ± 0.00	0.05 ± 0.00*	0.05
c22:2n6	0.06 ± 0.00*	0.05 ± 0.00	0.02
c22:5n3	0.10 ± 0.00	0.12 ± 0.00**	<0.01
c22:5n6	0.06 ± 0.00**	0.04 ± 0.00	<0.01
c23:0	0.90 ± 0.01*	0.86 ± 0.01	0.02
c24:0	4.14 ± 0.15**	2.58 ± 0.02	<0.01
c24:1n9	0.37 ± 0.05	0.78 ± 0.05**	<0.01
SFA	76.72 ± 1.11	76.09 ± 0.46	0.44
MUFA	42.51 ± 2.74**	20.35 ± 0.44	<0.01
PUFA	196.53 ± 1.28	198.43 ± 0.98	0.12
N6	83.23 ± 3.61**	53.62 ± 0.25	0.01
N3	113.31 ± 2.33	144.81 ± 0.73**	<0.01
N6/N3	0.74 ± 0.05**	0.37 ± 0.00	0.01
PUFA/SFA	2.56 ± 0.02	2.61 ± 0.00	0.06

Values (mean ± SD). *p < 0.05 and **p < 0.01.

##### 3.1.3.3 Analysis of the flavonoid composition of forage grasses

As shown in Table 5, the flavonoids in the forage grasses of the HG and HH groups differed greatly due to the differences in region. The apigenin, chrysin, dihydrokaempferol, and eriodictyol were considerably higher (*p <* 0.05) while the ferulic acid, naringin, and liquiritigenin were considerably lower (*p <* 0.05) in HG than in HH group.

**Table 5:** Regional variations in flavonoid and monosaccharide profiles of natural forage grasses.

Parameters	HG	HH	*p*-value
Flavonoid (mg/100 g)			
Apigenin	0.37 ± 0.00**	0.15 ± 0.00	<0.01
Biochanin A	0.15 ± 0.00**	0.03 ± 0.00	<0.01
Butin	0.0001 ± 0.00	0.0002 ± 0.00*	0.02
Catechin	0.04 ± 0.01	0.11 ± 0.00**	<0.01
Chrysin	0.004 ± 0.00**	0.001 ± 0.00	<0.01
Daidzein	0.03 ± 0.00**	0.02 ± 0.00	<0.01
Daidzin	0.01 ± 0.00	0.02 ± 0.00**	<0.01
Dihydrokaempferol	0.03 ± 0.00**	0.01 ± 0.00	<0.01
Epicatechin	0.06 ± 0.00*	0.04 ± 0.01	0.02
Epigallocatechin	0.12 ± 0.01**	0.03 ± 0.00	<0.01
Eriodictyol	0.67 ± 0.00	0.76 ± 0.01**	<0.01
Gallocatechin	0.012 ± 0.00*	0.004 ± 0.00	0.03
Genistein	0.33 ± 0.00**	0.14 ± 0.00	<0.01
Genistin	0.07 ± 0.00	0.30 ± 0.02**	<0.01
Glycitein	0.02 ± 0.00**	0.01 ± 0.00	<0.01
Glycitin	0.01 ± 0.00	0.02 ± 0.00**	<0.01
Isoliquiritigenin	0.001 ± 0.00	0.003 ± 0.00**	<0.01
Isorhamnetin	0.14 ± 0.00	0.20 ± 0.00**	<0.01
Vitexin	0.31 ± 0.00	0.90 ± 0.01**	<0.01
Kaempferide	0.01 ± 0.00	0.03 ± 0.00**	<0.01
Kaempferol	0.16 ± 0.00**	0.05 ± 0.00	<0.01
Liquiritigenin	0.0003 ± 0.00	0.0010 ± 0.00**	<0.01
Luteolin	1.64 ± 0.03**	0.91 ± 0.02	<0.01
Luteolin 7-O-glucoside	0.67 ± 0.06**	0.40 ± 0.02	0.01
Myricetin	0.08 ± 0.00**	0.01 ± 0.00	<0.01
Naringenin	0.08 ± 0.00**	0.01 ± 0.00	<0.01
Naringin	0.001 ± 0.00	0.004 ± 0.00**	<0.01
p-Coumaric acid	0.50 ± 0.00	0.74 ± 0.00**	<0.01
Phenylalanine	2.69 ± 0.01	2.91 ± 0.08*	0.04
Prunetin	0.49 ± 0.01**	0.14 ± 0.01	<0.01
Quercetin	0.08 ± 0.01	0.27 ± 0.01**	<0.01
Quercetin 3-glucoside	1.35 ± 0.03*	1.22 ± 0.05	0.04
Quercitrin	0.13 ± 0.02	0.38 ± 0.02**	<0.01
Rutin	1.91 ± 0.06**	0.19 ± 0.02	<0.01
Sakuranetin	0.22 ± 0.00**	0.01 ± 0.00	<0.01
Taxifolin	0.03 ± 0.00**	0.01 ± 0.00	<0.01
Sugar (mg/100 g)			
Cellobiose	10.28 ± 0.00**	7.27 ± 0.09	<0.01
Trehalose	14.16 ± 0.21	41.63 ± 0.59**	<0.01
Sucrose	1776.08 ± 7.72**	990.53 ± 5.62	<0.01
Maltose	45.84 ± 0.30**	39.56 ± 0.11	<0.01
Phenylglucoside	1.43 ± 0.00	4.21 ± 0.04**	<0.01
2-Deoxy-D-ribose	0.86 ± 0.00	3.56 ± 0.00**	<0.01
D-Xylulose	0.84 ± 0.01	1.07 ± 0.03**	<0.01
D-Xylose	7.41 ± 0.03	9.71 ± 0.07**	<0.01
Xylitol	0.74 ± 0.01	0.92 ± 0.00**	<0.01
D-Sorbitol	2.56 ± 0.04	14.90 ± 0.20**	<0.01
Barium D-ribose-5-phosphate	1.93 ± 0.01**	1.64 ± 0.05	0.01
D-Mannose	4.52 ± 0.08	5.25 ± 0.17**	0.01
Levoglucosan	7.25 ± 0.05	15.58 ± 0.05**	<0.01
Inositol	68.56 ± 0.35**	43.96 ± 1.09	<0.01
D-Glucuronic acid	4.17 ± 0.04	5.65 ± 0.09**	<0.01
Glucose	1438.54 ± 15.50	1891.57 ± 44.80**	<0.01
D-Galacturonic acid	2.00 ± 0.01**	1.42 ± 0.03	<0.01
D-Galactose	19.65 ± 0.16	46.62 ± 0.16**	<0.01
L-Fucose	3.34 ± 0.04**	2.72 ± 0.06	<0.01
D-Fructose	1063.08 ± 13.51	1295.75 ± 0.90**	<0.01
D-Arabinose	6.51 ± 0.05	6.80 ± 0.07**	0.01
D-Arabinitol	8.84 ± 0.02	9.32 ± 0.01**	<0.01
D-Ribose	17.54 ± 0.13**	15.30 ± 0.01	<0.01
L-Rhamnose	2.16 ± 0.01	3.48 ± 0.04**	<0.01

Values (mean± SD). *p < 0.05 and **p < 0.01.

##### 3.1.3.4 Analysis of the monosaccharide composition of forage grasses

As shown in Table 5, the monosaccharide contents differed significantly between the HG and HH groups, with cellobiose, sucrose, maltose, barium D-ribose-5-phosphate, inositol, D-galacturonic acid, L-fucose, and D-ribose being significantly greater in the HG group relative to the HH group, while monosaccharides such as trehalose, D-mannose, glucose, and D-galactose were found to be more abundant in the HH group (*p <* 0.05).

#### 3.1.4 Non-targeted metabolomics analysis of forage grasses

##### 3.1.4.1 Identification and analysis of metabolites

The total ion chromatogram (TIC) of the QC samples was overlapped and compared with the spectra, as shown in Supplementary Figures S1A,B. The experimental results showed that the response intensities and retention times of the chromatographic peaks were essentially overlapped, suggesting that the variation caused by instrumental errors was minimal throughout the experimental process. The peaks obtained from the experimental sample extracts were analysed by principal component analysis (PCA), as shown in Supplementary Figures S2A,B. Since the R^2^ X of the ESI^+^ and ESI-modes were 0.509 and 0.557, respectively, the ESI^−^ mode was chosen for subsequent analysis to guarantee more reliable data. For further analysis of the two sample sets, partial least squares discriminant analysis (PLS-DA) and orthogonal partial least squares discriminant analysis (OPLS-DA) were used to analyze the denser and more accurate models between the two sets of samples, as shown in Supplementary Figures S2C,D. The results showed that the HH and HG groups were distributed on both sides of the PLS-DA and OPLS-DA scoring maps, respectively, and exhibited significant intra-group clustering and inter-group separation. The model was subsequently validated using a permutation test to prevent overfitting during the modeling process, and Supplementary Figures S1C,D provide the results of the permutation test for PLS-DA (R^2^X = 0.339, R^2^Y = 0.997, Q^2^ = 0.865) as well as for OPLS-DA (R^2^X = 0.339, R^2^Y = 0.997, Q^2^ = 0.78). As the replacement retention decreased, both the R^2^ and Q^2^ values of the stochastic model gradually decreased, indicating an absence of overfitting in the original model.

##### 3.1.4.2 Bioinformatics analysis of differential metabolites

Differential metabolites between the two groups of samples are illustrated in the volcano plot (Supplementary Figure S1E). These were identified using the criteria of VIP > 1.0 and *p* < 0.05, resulting in the identification of 64 differential metabolites in the two groups (Supplementary Table S2), of which 39 metabolites were up-regulated in the HH group compared to the HG group. Most of the up-regulated differential metabolites were lipids and lipoid molecules, organic oxides, benzene ring-type compounds, and nucleosides, nucleotides, and analogs, while the 25 down-regulated differential metabolites were mostly organic heterocyclic compounds, organic acids, and their derivatives.

**Figure 1:**
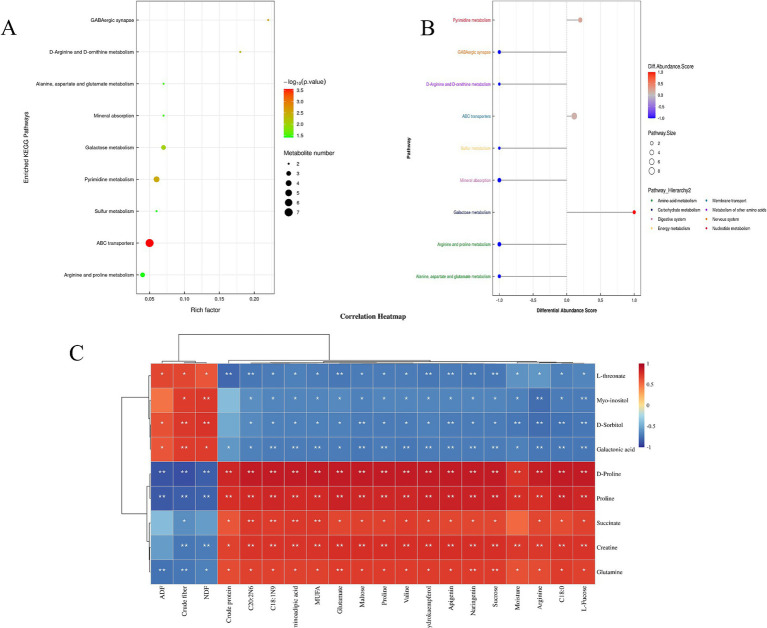
**(A)** Enrichment of the top 9 KEGG pathways for comparison between HH and HG; **(B)** Differential abundance scoring plot of differential metabolic pathways for HH vs. HG; **(C)** Heatmap of the correlation between pasture quality parameters and metabolomics analyses. *p < 0.05, **p < 0.01.

Figure 1A presents the enrichment analysis of the top 9 KEGG pathways, most of which are related to amino acid metabolism. Figure 1B provides a comparative analysis of pathways associated with metabolites that changed between the saline and common forages, showing that one metabolic pathway was up-regulated while five were down-regulated in the HH group compared to the HG group (DA > 0.5, *p* < 0.05). The up-regulated pathway was that of galactose metabolism, whereas the down-regulated metabolic pathways included GABAergic synapses, sulfur metabolism, mineral uptake, and arginine and proline metabolism, as well as the metabolism of alanine, aspartate, and glutamate. The up-regulated metabolites included sorbitol, galacturonic acid, and inositol, while the major down-regulated metabolites included glutamine, succinate, proline, creatine, and D-proline. Three of these metabolites, namely, proline, creatine, and D-proline, are involved in amino acid biosynthesis.

#### 3.1.5 Correlation between forage quality parameters and metabolomics

Figure 1C shows the correlations between pasture quality, amino acids and fatty acids in pastures and non-targeted metabolites, indicating the presence of significant correlations. C20: 2N6, C18: 1N9, aminoadipic acid, MUFA, glutamate, maltose, proline, valine, dihydrokaempferol, apigenin, naringenin, sucrose, moisture, arginine, C18: 0, and L-fucose were negatively correlated with the metabolites L-threonate, D-sorbitol, myo-inositol, and galactonic acid, while positively correlated with D-proline, proline, succinate, creatine, and glutamine. Crude protein was found to be negatively correlated with the metabolites L-threonate and galactonic acid while positively correlated with D-proline, proline, succinate, creatine, and glutamine. In addition, crude fiber and NDF were positively associated with the metabolites L-threonate, myo-inositol, D-sorbitol, and galactonic acid and negatively associated with D-proline, proline, succinate, creatine, and glutamine. ADF was positively correlated with L- threonate, D-sorbitol, and galactonic acid and negatively correlated with D-proline, proline, and glutamine. Due to the significant correlations between forage quality and metabolites, both these forage qualities and metabolites were incorporated in the joint analysis of meat and rumen microorganisms.

### 3.2 Analysis of the quality of Tibetan sheep meat and its metabolites

#### 3.2.1 Carcass quality analysis

As can be seen from Table 6, the difference in carcass quality between the HC and GC groups was not significant. Apart from the carcass weight in the HC group which was significantly greater than that of the GC group. Rib meat thickness, abdominal fat thickness, back fat thickness, and eye muscle area differed sightly between the two groups (*p <* 0.05).

**Table 6:** Analysis of meat quality, amino acid and fatty acid composition and content in the longest dorsal muscle of Tibetan sheep.

Parameters	GC	HC	*p*-value
Carcass quality			
Carcass weight(kg)	13.20 ± 0.35	13.73 ± 0.61	0.28
Rib fat thickness(cm)	2.13 ± 0.43	2.27 ± 0.17	0.49
Abdominal fat thickness(cm)	2.33 ± 0.36	2.57 ± 0.30	0.24
Backfat thickness(cm)	1.87 ± 0.28	2.01 ± 0.37	0.45
Eye muscle area(cm^2^)	13.16 ± 1.81	12.44 ± 2.06	0.54
Edible quality			
pH_45min_	6.89 ± 0.41	6.67 ± 0.34	0.10
pH_24h_	6.19 ± 0.33	5.98 ± 0.46	0.13
L*	32.70 ± 0.98	33.89 ± 0.69	0.17
a*	27.95 ± 0.64*	26.15 ± 0.44	0.02
b*	9.37 ± 0.53	14.40 ± 1.03**	0.01
Thawing loss (%)	10.77 ± 2.04	10.94 ± 2.96	0.84
Cooking loss (%)	29.36 ± 4.62	29.08 ± 3.60	0.85
Cooked meat percentage (%)	70.38 ± 5.80	68.51 ± 5.22	0.34
Shear force (N)	118.72 ± 45.37	115.75 ± 35.56	0.83
Hardness (N)	9.85 ± 3.92*	5.85 ± 2.10	0.05
Elasticity (mm)	5.00 ± 5.58	3.10 ± 0.42	0.17
Viscosity (mJ)	0.27 ± 0.11	0.40 ± 0.08*	0.04
Agglutinative (mJ)	6.26 ± 8.03	3.23 ± 1.87	0.14
Chewability (mJ)	8.95 ± 10.59	9.9 ± 5.25	0.75
Nutritional quality			
Protein (%)	21.97 ± 0.31	22.60 ± 0.36	0.08
Fat (%)	2.04 ± 0.47	2.65 ± 0.32	0.15
Moisture (%)	75.81 ± 0.22	74.00 ± 1.52	0.17
AA (mg/100 g)			
Aminoadipic acid	1.18 ± 0.07*	1.03 ± 0.06	0.05
Arginine	1091.84 ± 50.57*	969.69 ± 51.88	0.04
Asparagine	0.61 ± 0.02*	0.56 ± 0.02	0.05
Aspartate	144.67 ± 29.57*	70.57 ± 2.07	0.05
Citrulline	12.50 ± 0.01**	7.22 ± 0.10	<0.01
Creatine	173.79 ± 2.38*	163.33 ± 3.38	0.02
Creatinine	1.37 ± 0.00	1.53 ± 0.01**	<0.01
Cysteine	0.05 ± 0.00**	0.03 ± 0.00	<0.01
Cystine	41.09 ± 1.92	54.72 ± 1.00**	<0.01
Glutamate	34.04 ± 0.97	55.29 ± 2.54**	<0.01
Glutamine	18.50 ± 0.66**	13.99 ± 0.64	<0.01
Glycine	737.31 ± 3.11**	554.31 ± 6.88	<0.01
Hydroxyproline	0.58 ± 0.03	0.68 ± 0.02**	0.01
Proline	177.21 ± 7.45*	156.80 ± 7.03	0.03
Serine	228.62 ± 13.64*	183.57 ± 4.11	0.02
Spermidine	0.020 ± 0.00**	0.018 ± 0.00	<0.01
Taurine	12.47 ± 1.17	20.16 ± 2.83*	0.03
Tryptophan	1.84 ± 0.10*	1.62 ± 0.03	0.05
EAAs	1756.58 ± 81.12	1740.31 ± 7.40	0.76
NEAAs	3401.47 ± 68.37**	2931.35 ± 50.84	<0.01
TAAs	5158.05 ± 149.48*	4671.66 ± 58.24	0.02
FA (mg/100 g)			
C6:0	0.10 ± 0.01**	0.07 ± 0.01	0.01
C14:1N5	0.06 ± 0.00	0.09 ± 0.01**	0.01
C15:1N5	0.04 ± 0.00	0.05 ± 0.00*	0.02
C16:1N7	0.87 ± 0.02	1.43 ± 0.05**	<0.01
C17:0	1.19 ± 0.07	1.83 ± 0.13**	<0.01
C17:1N7	1.15 ± 0.13	1.69 ± 0.07**	0.01
C18:1N9	19.60 ± 1.65	26.29 ± 1.70**	0.01
C18:2TTN6	0.34 ± 0.01*	0.20 ± 0.03	0.02
C18:3N6	0.67 ± 0.03	0.88 ± 0.02**	<0.01
C20:3N6	1.60 ± 0.01*	1.26 ± 0.13	0.05
C20:4N6	7.72 ± 0.16	10.50 ± 0.91*	0.03
C20:5N3	7.34 ± 0.11**	3.61 ± 0.47	<0.01
C22:0	0.09 ± 0.01	0.14 ± 0.01**	0.01
C22:4N6	0.27 ± 0.00	0.46 ± 0.03**	0.01
C22:5N3	5.45 ± 0.15*	3.65 ± 0.60	0.03
C22:5N6	0.06 ± 0.00	0.08 ± 0.00*	0.02
C22:6N3	1.34 ± 0.11**	0.91 ± 0.10	0.01
C24:0	0.30 ± 0.04	0.48 ± 0.03**	0.01
SFA	33.17 ± 2.20	35.02 ± 2.37	0.38
MUFA	73.84 ± 3.85	88.84 ± 5.85*	0.03
PUFA	51.35 ± 1.83	49.86 ± 1.85	0.38
N6	24.34 ± 0.38	30.23 ± 2.30*	0.04
N3	26.90 ± 1.46**	19.50 ± 0.47	0.01
N6/N3	0.91 ± 0.04	1.55 ± 0.16*	0.02
PUFA/SFA	1.55 ± 0.05*	1.43 ± 0.04	0.03

Values (mean ± SD). *p < 0.05 and **p < 0.01.

#### 3.2.2 Meat quality analysis

##### 3.2.2.1 Sensory evaluation

As shown in Supplementary Figure S3, although the meat of the GC group scored higher than that of the HC group in terms of aroma, taste, texture, and overall acceptability, no significant difference was found between the two groups (*p <* 0.05).

##### 3.2.2.2 Edible quality analysis

Table 6 lists the differences in the edible quality of the meat of Tibetan sheep from different regions. The results showed that the a* value of the meat in the GC group was significantly higher than that of the HC group, while the b* value and stickiness were significantly lower than that of the HC group (*p <* 0.05). No significant differences between the two groups were found in shear force, thawing loss, and cooked meat percentage (*p <* 0.05).

##### 3.2.2.3 Nutritional quality analysis

In terms of nutritional quality (Table 6), the crude protein content was significantly higher in the HC group than that in the GC group, whereas there was no significant difference in moisture and fat contents between the two groups (*p <* 0.05).

#### 3.2.3 Targeted metabolomics analysis of Tibetan sheep

##### 3.2.3.1 Analysis of amino acid composition

Table 6 presents the differences in amino acid contents of the *longissimus dorsi* muscles of Tibetan sheep from different regions. From the table, it can be seen that the contents of amino acids such as arginine, aspartate, and citrulline were higher in the GC group compared to the HC group, while the contents of amino acids such as cystine, glutamate, and hydroxyproline were significantly higher in the HC group relative to the GC group (*p <* 0.05). Overall, the contents of non-essential amino acids (NEAAs) and total amino acids (TAAs) were significantly higher in the GC group than in the HC group (*p <* 0.05), while the difference between the two groups in the contents of essential amino acids (EAAs) was not significant (*p >* 0.05).

##### 3.2.3.2 Analysis of fatty acid composition

The differences in fatty acid contents between the GC and HC groups are shown in Table 6. Fatty acids such as C6: 0, C20: 3N6, C20: 5N3 were found in higher concentrations in the GC group, while the levels of others such as C14: 1N5, C17: 0, C18: 3N6 were considerably greater in the HC than in the GC group, while the contents of MUFA and N6 PUFAs, as well as the N6/N3 ratio, were higher in the HC group while the content of N3 PUFAs and the PUFA/SFA ratio were higher in the GC group (*p >* 0.05).

#### 3.2.4 Non-targeted metabolomics analysis of Tibetan sheep meat

##### 3.2.4.1 Metabolite identification and analysis

To investigate the effect of natural saline pasture on the *longissimus dorsi* muscles of Tibetan sheep, the samples were analyzed by UHPLC-QTOF-MS in the positive and negative ion modes. As shown in Supplementary Figures S4A,B, a comparison of the total ion flow plots of the QC samples by overlaying the plots revealed minimal variation due to instrumental errors throughout the experiment. The PCA score plots (Supplementary Figures S5A,B) show the differences between the two groups, from which it can be seen that the two groups did not show significant separation. Thus, the ESI^+^ mode was chosen for subsequent analyses as the R^2^X values in the ESI^+^ and ESI^−^ modes were 0.552 and 0.546, respectively. To further enhance intergroup separation, the two sample sets were further analyzed using PLS-DA and OPLS-DA, and Supplementary Figures S5C,D present the results of the analyses in the positive ion detection mode, with the samples exhibiting good intragroup aggregation and intergroup separation.

##### 3.2.4.2 Bioinformatics analysis of differential metabolites

**Figure 2:**
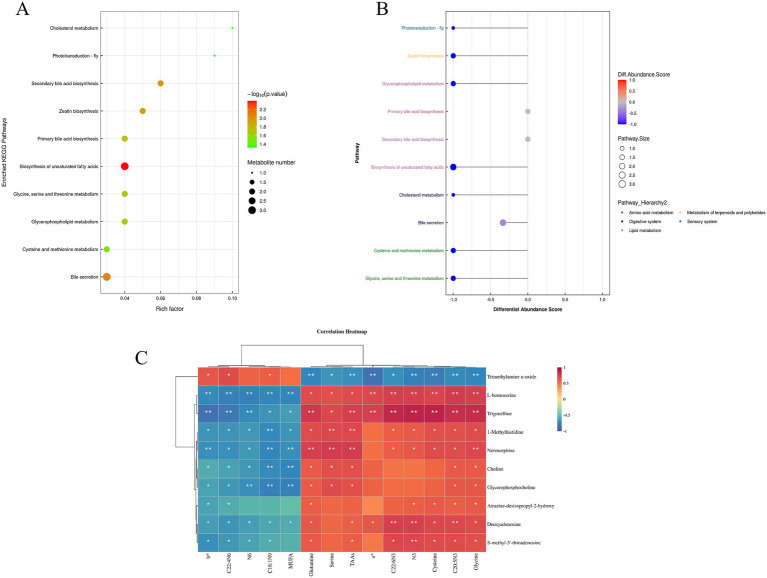
**(A)** Enrichment of the top 10 KEGG pathways compared between HC and GC groups. **(B)** Differential abundance score plot of differential metabolic pathways for HC VS GC; **(C)** Heatmap of correlation between meat quality parameters and metabolomics analysis. *p < 0.05, **p < 0.01.

The combined positive and negative ion modes identified 1,133 metabolites, of which 632 metabolites were identified in the positive ion mode and 501 metabolites in the negative ion mode. The volcano plot (Supplementary Figure S4E) shows 29 differential metabolites (VIP > 1.0, *p* < 0.05) obtained from the comparison between the GC and HC groups in the positive-ion detection mode, of which 13 were up-regulated and 16 down-regulated (Supplementary Table S3). Compared to the GC group, the up-regulated metabolites in HC belonged to nitrogen-containing heterocyclic compound classes, while the down-regulated metabolites were alkaloids and their derivatives, organic acids and their derivatives, benzene ring-type compounds, and nucleosides, nucleotides, and analogs. KEGG enrichment of the metabolites, shown in Figures 2A,B, revealed the top 20 metabolic pathways, most of which are associated with lipid metabolism, amino acid metabolism, and the sensory system. The results shown in Supplementary Table S4 indicate that one metabolic pathway was up-regulated and 10 metabolic pathways were down-regulated in the HC group compared with the GC group (DA > 0.5, *p* < 0.05). Specifically, the up-regulated metabolic pathway was the two-component system, and the key metabolite up-regulated was trimethylamine n-oxide, while the down-regulated metabolic pathways included those involved in glycine, serine, and threonine metabolism, glycerophospholipid metabolism, cysteine and methionine metabolism, cholinergic synapses, atrazine degradation, ether lipid metabolism, ABC transporters, sulfur metabolism, lysine biosynthesis, and histidine metabolism, with the major down-regulated metabolites including S-methyl-5′-thioadenosine, choline, glycerophosphocholine, L-homoserine, atrazine-desisopropyl-2-hydroxy, deoxyadenosine, and 1-methylhistidine.

#### 3.2.5 Correlation between meat quality parameters and metabolomics

The correlations between meat quality parameters, amino acids, fatty acids and non-target metabolites in meat are shown in Figure 2C. It is clear that there is a significant association between meat quality and muscle metabolism. It was found that b*, C22: 4 N6, and C18:1 N9 were positively correlated with the metabolite trimethylamine n-oxide while negatively correlated with the metabolites L- homoserine, trigonelline, 1-methylhistidine, normorphine, choline, glycerophosphocholine, atrazine- desisopropyl- 2-hydroxy, deoxyadenosine, and S-methy-5′-thioadenosine. N6 MUFA was negatively correlated with the metabolites L- homoserine, trigonelline, 1-methylhistidine, normorphine, choline, glycerophosphocholine, deoxyadenosine, and S- methy-5′-thioadenosine. Glutamine, serine, TAAs, a*, C22: 6 N3, N3, cysteine, C20: 5 N3, and glycine were negatively associated with the metabolite trimethylamine n-oxide, and glutamine, TAAs, C20: 5 N3, and glycine were positively associated with L-homoserine, trigonelline, 1-methylhistidine, normorphine, choline, glycerophosphocholine, atrazine-desisopropyl-2-hydroxy, deoxyadenosine, and S- methy-5′-thioadenosine. C22: 6 N3, N3, and cysteine were positively correlated with L-homoserine, trigonelline, 1- methylhistidine, normorphine, atrazine-desisopropyl-2-hydroxy, deoxyadenosine, and S-methy-5′-thioadenosine. Serine was positively associated with L-homoserine, trigonelline, 1-methylhistidine, normorphine, choline, and glycerophosphocholine and a* was positively correlated with L-homoserine, trigonelline, and deoxyadenosine. Therefore, the components that were co-analyzed with forage were b*, C22: 4 N6, N6, C18:1 N9, glutamine, TAAs, N3, cysteine, C20: 5 N3, and glycine and the metabolites trimethylamine n-oxide, L-homoserine, trigonelline, 1-methylhistidine, normorphine, deoxyadenosine, and S-methy-5′-thioadenosine.

### 3.3 Rumen characteristics

#### 3.3.1 Characterization of rumen fermentation

The results shown in Table 7 show that there are significant differences in pH and SCFAs in rumen fluid between the GC and HC. However, the contents of butyric acid, acetic acid, valeric acid, propionic acid, and TSCFAs are higher in HC group, but lower in GC group (*p <* 0.05). In addition, the pH and isobutyric acid concentration in GC group were significantly higher than in the HC group (*p <* 0.05).

**Table 7:** Effect of forage diets from different regions on the rumen fermentation characteristics and the level of rumen bacterial phylum and genus in Tibetan sheep.

Items	GC	HC	*p*-value
The rumen fermentation characteristics			
pH	7.46 ± 0.06**	7.25 ± 0.05	0.01
Acetic acid (ug/mL)	631.12 ± 17.86	940.58 ± 46.74**	<0.01
Propionic acid (ug/mL)	250.55 ± 15.13	340.36 ± 2.04**	0.01
Butyric acid (ug/mL)	247.11 ± 2.65	293.73 ± 18.46*	0.05
Isobutyric acid (ug/mL)	124.51 ± 1.72**	118.16 ± 1.62	0.01
Valeric acid (ug/mL)	54.70 ± 1.61	60.97 ± 2.69*	0.04
Isovaleric acid (ug/mL)	108.55 ± 0.56	111.62 ± 1.55	0.06
Hexanoic acid (ug/mL)	4.28 ± 0.53	5.19 ± 1.07	0.28
TSCFAs (ug/mL)	1420.80 ± 31.31	1870.61 ± 49.69**	<0.01
A/P	2.52 ± 0.08	2.76 ± 0.14	0.08
Phylum level (%)			
*Bacteroidota*	46.08 ± 3.40	53.22 ± 3.17**	<0.01
*Firmicutes*	34.44 ± 2.77**	29.28 ± 2.12	0.01
*Verrucomicrobiota*	7.77 ± 1.03*	6.27 ± 1.08	0.03
*Synergistota*	1.47 ± 0.65	2.91 ± 1.07*	0.02
*Proteobacteria*	1.99 ± 1.47	1.37 ± 0.52	0.36
*Patescibacteria*	1.70 ± 0.75	1.49 ± 0.78	0.65
*Spirochaetota*	1.18 ± 0.27	1.55 ± 0.42	0.10
*Cyanobacteria*	1.27 ± 0.39**	0.60 ± 0.29	0.01
*Fibrobacterota*	0.81 ± 0.67	0.98 ± 0.33	0.61
*Desulfobacterota*	1.02 ± 0.41	0.62 ± 0.14	0.07
Genus level (%)			
*Rikenellaceae_RC9_gut_group*	12.49 ± 2.63	13.25 ± 3.08	0.66
*F082*	12.67 ± 0.83**	7.33 ± 0.26	<0.01
*Prevotella*	8.56 ± 0.13	8.36 ± 0.74	0.54
*WCHB1-41*	4.88 ± 0.07**	3.91 ± 0.23	<0.01
*Prevotellaceae_UCG-003*	2.65 ± 1.01	4.13 ± 1.22*	0.05
*Bacteroidales_UCG-001*	2.57 ± 0.28	4.40 ± 0.19**	<0.01
*[Eubacterium]_coprostanoligenes_group*	2.34 ± 0.10	3.12 ± 0.07**	<0.01
*Christensenellaceae_R-7_group*	2.35 ± 0.12	2.17 ± 0.40	0.33
*Bacteroidales_BS11_gut_group*	0.88 ± 0.41	0.94 ± 0.07	0.75
*Fretibacterium*	1.31 ± 0.17	2.71 ± 0.31**	<0.01

Values (mean ± SD). *p < 0.05 and **p < 0.01.

#### 3.3.2 Analysis of rumen microbiota composition

**Figure 3:**
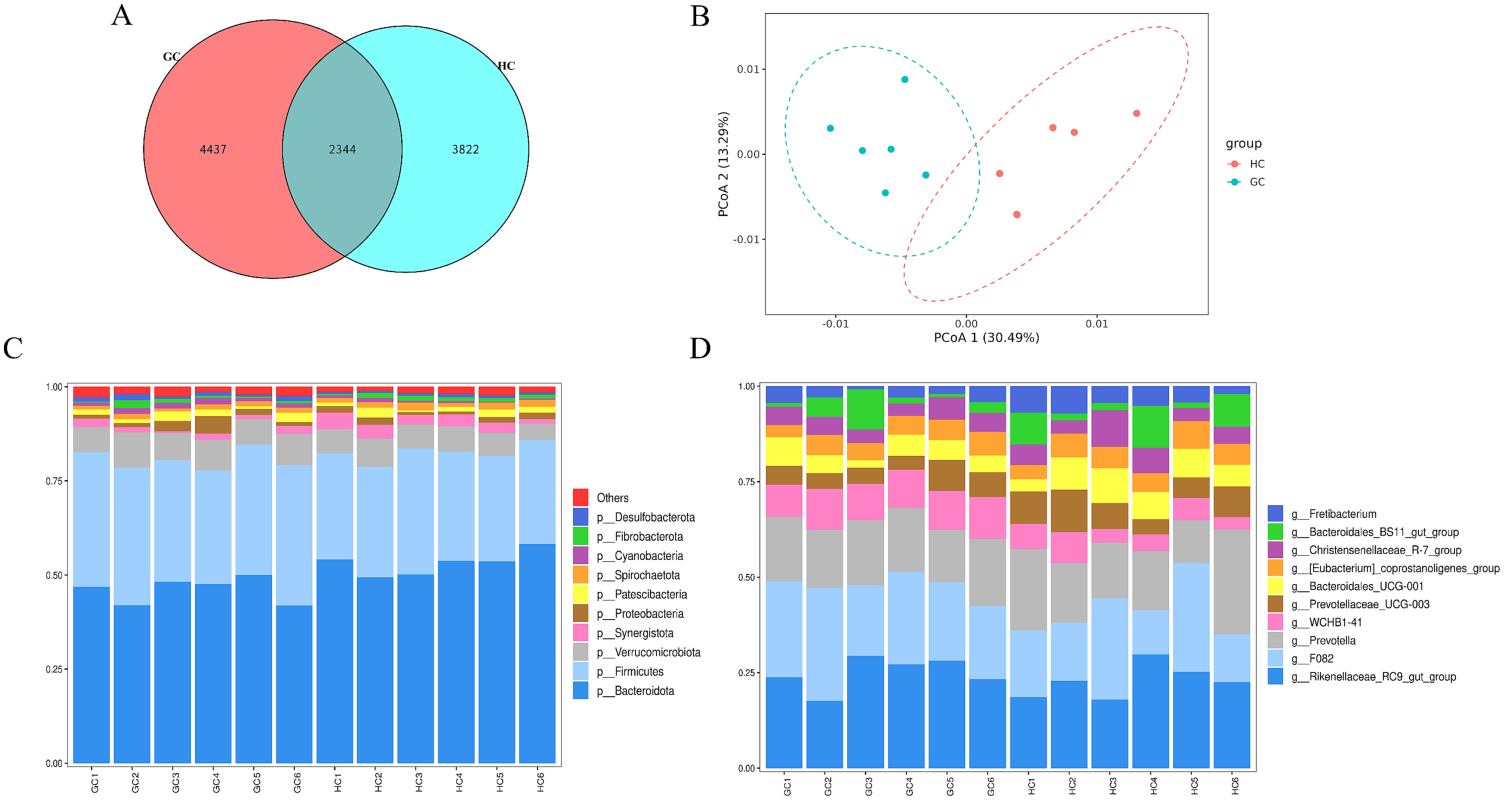
**(A)** Vene plot of ASVs of rumen microorganisms from GC and HC groups; **(B)** PCoA plot of total rumen microbial samples; relative abundance of bacterial communities at phylum **(C)** and genus **(D)** levels in both groups.

A total of 10,603 ASVs were identified in both the GC and HC groups, of which 4,437 and 3,822 specific ASVs were observed in the GC and HC groups, respectively, as shown in Figure 3A. Comparing the *α*-diversity indices among the three groups, as shown in Supplementary Table S5, it was found that the Shannon and Simpson indices in the GC group were significantly higher than those in the HC group, suggesting that the diversity of microbiota in the GC group was significantly higher than that in the HC group. Subsequent analysis using the PCoA plots (Figure 3B) showed significant separation of the bacterial communities of the two groups, as seen in Figure 3C. At the phylum level, the predominant phyla in the rumen were *Bacteroidetes* and *Firmicutes*. At the genus level (Figure 3D), the dominant taxa were *Rikenellaceae RC9 gut group*, *F082*, and *Prevotella*. Table 7 lists the main differences between the rumen bacteria of the GC and HC groups at the phylum and genus levels. The abundance of the *Firmicutes*, *Verrucomicrobiota*, and *Cyanobacteria* phyla was significantly higher (*p <* 0.05) in the GC group relative to the HC group, while the abundance of *Bacteroidota* and *Synergistot*a was higher (*p <* 0.05) in the HC group. At the genus level, the abundance of *F082* and *WCHB1-41* was significantly greater (*p <* 0.05) in the GC group compared with the HC group, while the abundance of *Prevotellaceae_UCG-003*, *Bacteroidales_UCG-001, [Eubacterium]_coprostanoligenes_group*, and *Fretibacterium* was significantly lower (*p <* 0.05) than in the HC group.

#### 3.3.3 Correlation between rumen microorganisms and short-chain fatty acids in the rumen

**Figure 4:**
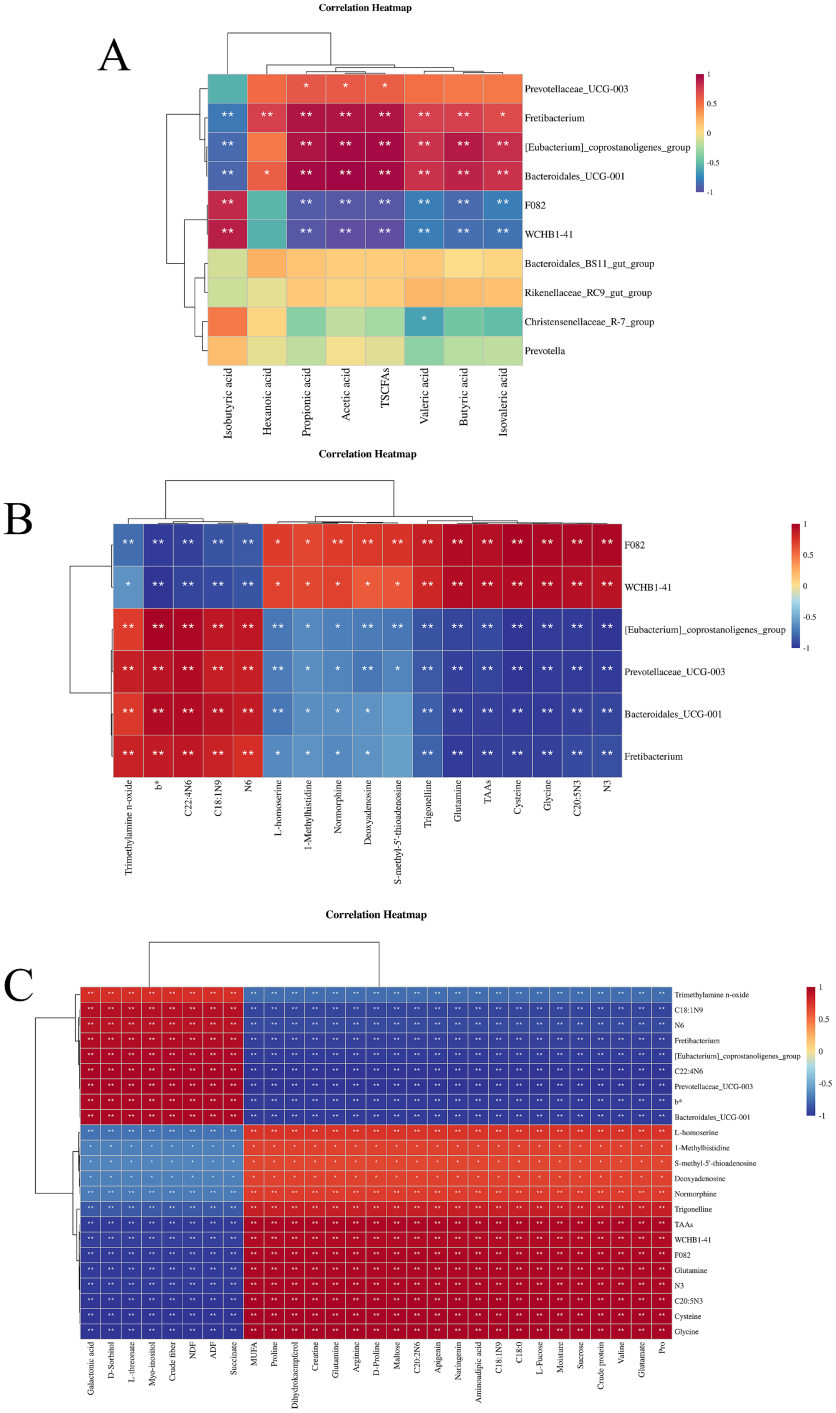
**(A)** Heat map of correlation between rumen microorganisms and short-chain fatty acids; **(B)** Heat map of correlation between muscle metabolites and rumen microorganisms; **(C)** Clustered heat map of correlation of metabolites with feed, meat quality and rumen microorganisms. *p < 0.05, **p < 0.01.

The correlation between rumen microbes and short-chain fatty acids is shown in Figure 4A. It can be seen that the rumen microbes *Fretibacterium*, *[Eubacterium]_coprostanoligenes_group,* and *Bacteroidales_UCG-001* were negatively associated with isobutyric acid, and positively with hexanoic acid, acetic acid, propionic acid, TSCFAs, butyric acid, valeric acid, and isovaleric acid. *F082*, and *WCHB1-41* were positively correlated with isobutyric acid while negatively correlated with propionic acid, acetic acid, TSCFAs, valeric acid, butyric acid, and isovaleric acid, while *Prevotellaceae_UCG-003* was positively correlated with propionic acid, acetic acid and TSCFAs and *Christensenellaceae_R-7_group* was negatively correlated with valeric acid.

#### 3.3.4 Correlation between rumen microorganisms and meat quality and metabolites in the longissimus dorsi muscle

The associations between muscle metabolites and rumen microorganisms are shown in Figure 4B. It can be seen from the figure that these were significantly associated. Specifically, trimethylamine n-oxide, b*, C22: 4 N6 C18:1 N9, and N6 were negatively correlated with the rumen microbes *F082* and *WCHB1-41*, while positively correlated with *[Eubacterium]_coprostanoligenes_group, Prevotellaceae_UCG-003, Bacteroidales_UCG-001*, and *Fretibacterium*. L-homoserine, 1-methylhistidine, normorphine, deoxyadenosine, trigonelline, glutamine, TAAs, cysteine, glycine, C20: 5 N3, and N3 were positively correlated with *F082* and *WCHB1-41* while negatively correlated with *[Eubacterium]_coprostanoligenes_group, Prevotellaceae_UCG-003, Bacteroidales_UCG-001*, and *Fretibacterium*. S-methy-5′-thioadenosine was positively correlated with *F082* and *WCHB1-41* while negatively correlated with *[Eubacterium]_coprostanoligenes_group* and *Prevotellaceae_UCG-003*.

### 3.4 Conjoint analysis

The correlation clustering heatmap shown in Figure 4C depicts the relationships among pasture, the *longissimus dorsi* muscle, and rumen microorganisms, from which it can be seen that there are significant associations among them. Crude fiber, NDF, ADF and the metabolites galactonic acid, D-sorbitol, L- threonate, myoinositol. Succinate in the natural forage were positively correlated with trimethylamine n-oxide, C18:1 N9, N6, C22: 4 N6, and b* in the muscle, and the rumen microorganisms *Fretibacterium, [Eubacterium]_coprostanoligenes_group, Prevotellaceae_UCG-003*, and *Bacteroidales_UCG-001,* while negatively correlated with L-homoserine, 1-methylhistidine, S-methy-5′- thioadenosine, deoxyadenosine, normorphine, trigonelline, TAAs, glutamine, N3, C20: 5 N3, cysteine, glycine and the rumen microbes *F082* and *WCHB1-41*. MUFA, dihydrokaempferol, arginine, maltose, C20: 2 N6, apigenin, naringenin, aminoadipic acid, C18: 1 N9, C18: 0, L-fucose, moisture, sucrose, crude protein, valine, glutamate, proline, creatine, glutamine, and D-proline in pasture were negatively correlated with trimethylamine n-oxide, C18:1 N9, N6, C22: 4 N6, and b* in muscle and the rumen microorganisms *Fretibacterium*, *[Eubacterium]_coprostanoligenes_group*, *Prevotellaceae_UCG-003*, and *Bacteroidales_UCG-001*, while positively correlated with L-homoserine, 1-methylhistidine, S-methy-5′-thioadenosine, deoxyadenosine, normorphine, trigonelline, TAAs, glutamine, N3, C20: 5 N3, cysteine, and glycine in muscle and the rumen microorganisms *F082* and *WCHB1-41.*

## 4 Discussion

Forage quality exerts significant effects on the meat quality of ruminants (Zhang and Dai, 2023). Previous studies have demonstrated that Leymus chinensis, with its superior nutritional value compared to corn straw, significantly enhances the growth performance of Holstein bull calves (Yu, 2008). Through comparative analysis of saline-alkali forage versus non-saline-alkali pasture, Ma et al. (2024) demonstrated that saline-alkali grass exhibits superior apparent quality. When fed to Tibetan sheep, this forage enhances meat quality by mitigating pH decline, increasing unsaturated fatty acid content, and reducing thawing loss. However, there have been few reports on the effects of saline-alkali forage on the meat quality and rumen microbiota of Tibetan sheep. This study, integrating plant sampling and animal experiments, investigated the relationship between the quality of saline-alkali forage, the rumen microbiota of Tibetan sheep, and the meat quality. The results revealed that natural saline-alkali forage can indeed significantly influence the composition of rumen microbiota and the meat quality of Tibetan sheep.

### 4.1 The differences in the quality and metabolites of natural forages from different regions

In recent years, there has been extensive research on the physiological mechanisms underlying plant adaptations to saline and alkaline stress. The results of these studies have demonstrated the multiplicity and complexity of the adaptive mechanisms, triggering changes in osmoregulatory substances, such as the soluble sugars sucrose and maltose, and proline. Soluble sugars play an important role in adaptive buffering and maintaining the osmotic pressure balance when plants are subjected to saline and alkaline stresses (Mostofa et al., 2015; Han et al., 2016). It has been reported that in *Suaeda salsa,* salt stress leads to significant increases in the levels of both sucrose and maltose, providing resistance to unfavorable environmental conditions (Wang X. et al., 2021). The levels of sucrose and proline levels have been observed to increase in wheat leaves under high salt stress (Guo, 2015). Similarly, alfalfa leaves adapt to high alkalinity and salinity by accumulating soluble sugars (Wang Y. et al., 2021). Proline is considered an osmotic regulator and accumulates significantly in numerous plants under saline conditions, including cotton, the Australian wild rice (*Oryza australiensis domin*), and alfalfa (*Medicago sativa* L.) to balance osmotic pressure and adapt to saline and alkaline stress (Nguyen et al., 2021; Dong et al., 2023; Chen et al., 2024). In the HG group in the present study, significant accumulation of proline, sucrose, and maltose was observed, suggesting that these small-molecule organic compounds play an important osmoregulatory role in adaptation to salinity pasture grasses. Also of interest are studies proposing that plants growing in saline areas maintain water uptake by the accumulation of osmotic agents to reduce osmotic potential and maintain the solubilization pressure (Lu et al., 2021). This is consistent with the present results.

The quality of pasture affects the growth and development of ruminants that depend on that pasture as their entire food source for extended periods, and thus also on the yield and quality of the animal products (Zhang et al., 2023; Zhang et al., 2023). This indicates the importance of evaluating nutritional quality indicators in forage. It has been reported that the crude protein (CP) content of pasture is positively correlated with its nutritional quality, whereas higher contents of acid detergent fiber (ADF) are associated with reduced digestibility and consequent poor animal utilization and nutrient uptake; similarly, higher contents of neutral detergent fiber (NDF) reduce the palatability of the pasture (Allen, 2000; Elgersma and Søegaard, 2016; Sun et al., 2020; Zha et al., 2022). Therefore, high protein and low fiber contents are significant indicators of high-quality forage, which also highlights the high quality of the forage in the HG group.

Saline stress leads to excessive production of reactive oxygen species (ROS) by the plant, and ROS accumulation induces oxidative stress and cellular damage (Li C. et al., 2022). In this context, the accumulation of flavonoids, secondary metabolites widely distributed in plants, contributes to the enhancement of the antioxidant capacity of plants (Ma et al., 2020). Flavonoids mitigate plant damage caused by abiotic stresses by scavenging free radicals and preventing the accumulation of ROS (Hasanuzzaman, 2020). It has been shown that when plants are subjected to saline stress, flavonoid accumulate in large quantities (Liu L. et al., 2024; Lv et al., 2024; Ma et al., 2020). In this study, however, the contents of most flavonoids such as apigenin, naringenin, dihydrocannabinol, and epicatechin, were significantly higher in the HG group relative to HH, which may indicate that flavonoid accumulation is an important means of coping with oxidative stress in pasture grasses of the HG group.

In the present study, the untargeted metabolomics results highlighted enrichment in pathways associated with amino acids, specifically, pathways involved in the metabolism of alanine, aspartic acid, and glutamic acid, as well as the metabolism of arginine and proline, both of which have been reported in previous studies (Li, 2021; Zheng et al., 2022; Duan et al., 2023). In addition, Li (2021) found that salt treatment had a greater effect on sulfur metabolism in Nitraria sibirica Pall, while Qian et al. (2023) observed an association between saline stress and the glyoxylate and dicarboxylate metabolic pathways. This is consistent with the metabolic pathway enrichment results found in the salt-tolerant forage of the HG group. In addition, the differential metabolites derived from metabolic pathways up-regulated in the HG group were found to consist of organic acids and their derivatives, succinate, proline, and glutamine. Many plants secrete large amounts of organic acids under saline and alkaline stress, and these organic acids can maintain the stability of the intracellular pH and ionic balance by neutralizing bases as well as reducing excess levels of toxic ions (Chen, 2022). The important role of succinate as an organic acid in plant tolerance to salinity is well-documented (Yang et al., 1994; Wang X. et al., 2011). In addition, Li Z. et al. (2022) found that exogenous application of spermine responded to salt stress by enhancing the synthesis of various amino acids, such as glutamine, in plants. Similarly, under salt stress, the durum wheat root system accumulates glutamine and proline in the cytoplasm to regulate its osmotic pressure (Carillo, 2017). Therefore, we suggest that the accumulation of organic acids and their derivatives, succinate, proline, and glutamine, is an important physiological response of HG pasture grasses to saline and alkaline stress.

Numerous studies have shown that forage has a significant effect on meat quality. Ding et al. (2021) found that dietary supplementation with Allium mongolicum Regel and its extracts significantly reduced both drip loss and cooking loss in lamb meat, improving the meat quality. The addition of dehydrated leguminous-based forage to the diet provides broilers with a more favorable ratio of polyunsaturated fatty acids (Ponte et al., 2008), while dietary supplementation with *Plantago lanceolata* L. and *Allium sativum* improves fatty acid profiles in sheep, resulting in lower levels of saturated fatty acids and higher contents of polyunsaturated fatty acids, which can help to promote sheep growth and the production of lean lamb meat (Redoy et al., 2020). In this study, the use of saline pasture as feed for ruminants has significant advantages in various aspects and its effect on meat quality can be illustrated by the assessment of meat quality parameters, muscle metabolism, and rumen microbial compositions.

### 4.2 The differences in the meat quality of Tibetan sheep from different regions

The color of meat is an important factor influencing consumer preference (Suman et al., 2023). In general, meat with higher a* values and lower L* and b* values shows better color (Ma et al., 2024). In this study, the GC group had higher a* and lower b* values compared with those in the HC group, while L* did not differ significantly between the two groups. Liu et al. (2021) reported that increases in the redness index a* of muscle may be due to the oxidation of myoglobin resulting in bright red oxygenated myoglobin. However, both myoglobin and oxymyoglobin can be oxidized directly to brown myoglobin with a high iron content (Su et al., 2022). This reaction can adversely affect the color of the meat. De Brito et al. (2017) observed higher a* values in longissimus dorsi muscles of lambs with high antioxidant concentrations. In contrast, changes in b* values are associated with lipid oxidation, with the balance between chemical components such as unsaturated fatty acids and antioxidants determining the extent of lipid oxidation (Ponnampalam et al., 2017; Zhang et al., 2022a). Zhang et al. (2022a) observed that reduced antioxidant capacity in Tibetan sheep muscles was associated with higher b* values. Nieto et al. (2010) reported that dietary supplements could inhibit cell membrane oxidation by the introduction of natural antioxidants into phospholipid membranes. Furthermore, Ma et al. (2024) found that the presence of large amounts of antioxidants in saline pasture effectively prevented the oxidation of fatty acids in the muscles of Tibetan sheep. In the present study, we found higher contents of crude flavonoid polyphenols in the HG saline pasture group. Therefore, it was hypothesized that the increase in the a* value and decrease in the b* value observed in the GC group might be due to the presence of antioxidants such as flavonoids in the HG pasture. After intake of the flavonoid-rich pasture by the sheep, the phenolic hydroxyl and carbonyl groups in the flavonoid molecules can combine with metal ions to block the production of free radicals and thus enhance the antioxidant capacity of the muscles, while increasing the activity of high-iron myoglobin reductase, thus slowing the oxidation of myoglobin (Chen et al., 2020). Thus reducing the yellowness (b* value) and increasing the redness (a* value) of the muscle are associated with a better meat color. In addition, analysis of the correlations between Tibetan lamb quality indicators and muscle metabolites (Figure 2C) showed that the amino acid metabolism-enriched metabolites L-homoserine, S-methyl-5′-thioadenosine, and choline were positively correlated with the a* values and negatively correlated with the b* values. It is therefore hypothesized that the increased concentrations of L-homoserine and S-methyl-5′-thioadenosine in the GC group may promote the accumulation of antioxidants by modulating cysteine and methionine metabolic pathways, resulting in a better coloration of the meat in the GC group. In summary, antioxidant components, such as flavonoids, in saline pastures and amino acid metabolism in muscle tissues both influence muscle color.

### 4.3 The differences in the metabolites of Tibetan sheep meat from different regions

The type and contents of amino acids in muscle have an important influence on the flavor and nutritional value of meat, and the concentrations of some amino acids are strongly linked to metabolism. For example, serine is involved in the synthesis of methionine, glycine, and cysteine, and plays a role in lipid metabolism and the immune system, while glycine and serine are sweet amino acids, and thus contribute to the flavor of the lamb meat (Su et al., 2022). Cysteine is a precursor of glutathione and taurine, both of which have strong antioxidant capacities, and increases in the cysteine concentration can thus contribute to the production of antioxidant substances, which can effectively prevent the oxidation of fatty acids (Medina et al., 2022; Liu J. et al., 2024). Indeed, the concentrations of cysteine, serine, and glycine were observed to be higher in the *longissimus dorsi* muscles of the GC group through the up-regulation of glycine, serine, and threonine metabolism, as well as cysteine and methionine metabolism. Elevated cysteine concentrations contribute to the muscle antioxidant capacity, which may also be a reason for the high a* and low b* values in the *longissimus dorsi* muscles of the Tibetan sheep in the GC group. We also found that the TAAs content in the GC group was considerably greater than that in the HC group (*p* < 0.05); this may be related to the higher crude protein content and ideal amino acid ratio of the forage in the HG group, or it may have resulted from the saline forage acting as a substrate for the fermentation by rumen bacteria, which improved the rumen fermentation pattern and thus promoted the deposition of amino acids in the muscle.

The content and composition of fatty acids in meat affect both the flavor and quality of the meat (Xu et al., 2023). Unsaturated fatty acid concentrations affect meat flavor and nutritional value (Long et al., 2022). Monounsaturated fatty acids are associated with reduced risk of cardiovascular disease (Pinheiro et al., 2023). Polyunsaturated fatty acids play an important role in counteracting obesity and inflammation and are beneficial to human health (Liu J. et al., 2024). In particular, N3 PUFA are functional fatty acids that are associated with immune regulation (De Brito et al., 2017). In this study, the levels of MUFA and N6 PUFA and the values of N6/N3 were lower in the GC group than in the HC group, whereas the levels of N3 PUFA were higher than those in the HC group. Similar results were obtained in a study by Ponnampalam et al. (2017), which were attributed to the fact that the enzymes used for the elongation and desaturation processes are the same for both groups of fatty acids, N6, and N3, and therefore need to be shared; however the conversion of alpha-linolenic acid (ALA) by the enzymes to EPA and DHA appeared to be inhibited by the levels of N6 fatty acids present in the muscle tissue.

### 4.4 The differences in the rumen microbiota communities of Tibetan sheep from different regions

The *α*-diversity indices (Shannon and Simpson indices) indicated higher bacterial diversity and abundance in the GC group, suggesting that the type of pasture had a direct effect on the rumen microbial composition in Tibetan sheep. In the present study, there was a significant difference in the rumen microbial compositions of Tibetan sheep fed saline and common pasture. At the phylum level, it was found that *Bacteroidota* and *Firmicutes* were the predominant phyla in the rumen, which is consistent with the results of the dominant phyla previously identified in the rumen of Tibetan sheep (Cui et al., 2019). Moreover, the abundance of *Firmicutes* was higher in the GC group compared to the HC group, whereas the abundance of the *Bacteroidota* was significantly lower. *Bacteroidota* are involved in the degradation of carbohydrates and the production of acetate and propionate (Ahmad et al., 2020), while *Firmicutes* are associated with the degradation of cellulose and hemicellulose (Crisol-Martínez et al., 2017), however, *Firmicutes* are likely to attach to pasture particles with low ADF concentrations (Gharechahi et al., 2021). In this study, it was found that the ADF content was lower in the HG saline pasture, suggesting that the abundance of *Firmicutes* in the rumen of Tibetan sheep in the GC group may have been influenced by the low ADF content in the HG pasture. At the genus level, *Prevotella* plays an important role in the efficient utilization of hemicellulose, as well as in protein and peptide metabolism (Zhang et al., 2022a). *Prevotellaceae_UCG-003* (Liu J. et al., 2024) is associated with enhanced plant fiber digestion. The higher abundance of *Prevotellaceae_UCG-003* in the HC group may have been related to the higher fiber content in the diet of Tibetan sheep in the HC group. In addition, Du et al. (2023) showed that *Prevotellaceae_UCG-003* is associated with fatty acid synthesis, suggesting that the present finding of higher levels of MUFA and N6 PUFA in the HC group may have been linked to the higher abundance of *Prevotellaceae_UCG-003* in the HC group. The abundance of *F082* and *WCHB1-41* was significantly higher in the GC group than in the HC group (*p* < 0.05). However, there are few reports on the role of these two microorganisms in the rumen.

Previous studies have shown that changes in feed type can not only alter the availability of fermentation substrates but also induce changes in the internal environment of the rumen, affecting the rumen pH (Ghimire et al., 2017). In the present study, the pH of the rumen of Tibetan sheep in the GC group was higher than that in the HC group (*p* < 0.05), consistent with the results of Wang C. et al. (2011). Increased rumen concentrations of VFA are associated with reduced rumen pH (Ahmad et al., 2020). A higher rumen pH in the GC group relative to that in the HC group together with a lower total short-chain fatty acid concentration was also observed here. Short-chain fatty acids are the end-products of feed digestion and are the main source of energy for ruminant growth (Gharechahi et al., 2021). In the present study, we detected significantly higher levels of isobutyric acid in the GC group relative to the HC group, whereas the levels of propionic acid, acetic acid, valeric acid, and butyric acid were higher in the HC group. Guo et al. (2024a) reported that isobutyric acid in the rumen is associated with the catabolism of proteins and branched-chain amino acids, suggesting that the elevated concentration of total amino acids in the GC group was not only associated with high levels of total amino acids in saline pastures but also with the protein utilization efficiency of the rumen microbiota. The correlation analysis of the rumen microbial community structure with fermentation parameters (Figure 4A) revealed that isobutyric acid was positively correlated with *F082* and *WCHB1-41.* W. Wang W. et al. (2024); Wang H. et al. (2024) found that *WCHB1-41* may regulate the synthesis of rumen microbial proteins in yaks, and it was thus hypothesized that both *F082* and *WCHB1-41* may be associated with the utilization of rumen microbial proteins in Tibetan sheep. Acetic acid production in the rumen is closely related to fiber degradation (Fan et al., 2021). Deusch et al. (2017) also found a higher proportion of acetate and propionate in fiber-rich diets, and interestingly the correlation analysis in Figure 4A shows that both acetic acid and propionic acid were positively correlated with *Prevotellaceae_UCG-003*. Thus, the higher proportion of acetic and propionic acids in the HC group contributed to the growth of cellulolytic bacteria and thus to the degradation of the high fiber content of the feed.

### 4.5 The relationship among natural forages, rumen microbiota of Tibetan sheep, and the meat quality of Tibetan sheep

This study utilized the Pearson correlation coefficient to investigate the relationships among the quality of natural mixed forages, the rumen microbiota of Tibetan sheep, and the meat quality of Tibetan sheep. The analysis results indicate that the changes in the rumen microbiota of Tibetan sheep are regulated by natural mixed forages, and there is also a significant correlation between the rumen microbiota and the meat quality of Tibetan sheep. Notably, the correlation analysis shown in Figure 4C indicated that the crude protein in forage and specific forage metabolites, namely, glutamine, proline, D-proline, and creatine, were positively correlated with the TAAs content in the muscle, suggesting that the abundant crude protein content, the enrichment of amino acid metabolites in the forage, as well as up-regulation of amino acid metabolic pathways, affects the TAAs concentration in muscle. Guo et al. (2024b) reported that the crude protein content in forage was significantly correlated with the amino acid metabolites in yaks, which is consistent with our findings. Additionally, N3 PUFA in muscle exhibited a significant positive correlation with forage fatty acids (MUFA, C20:2 N6, C18:1 N9, C18:0), whereas N6 PUFA showed the opposite trend. This indicates that the fatty acid composition of natural mixed forage can significantly influence the fatty acid content in Tibetan sheep muscle, which aligns with previous reports (Jerónimo et al., 2009; Madziga et al., 2024).

**Figure 5:**
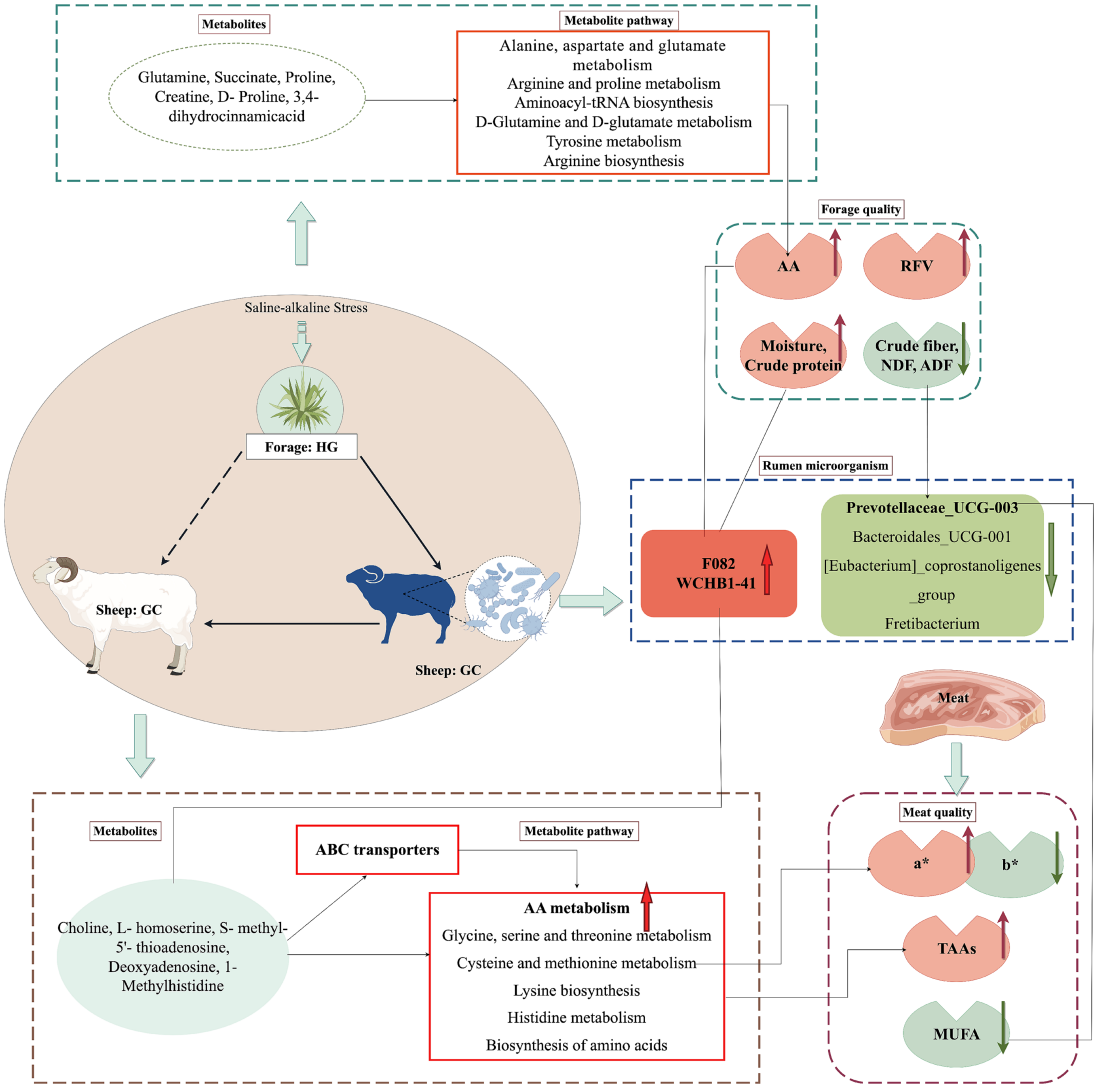
Hypothesised pathways and potential mechanisms associated with changes inforage quality, muscle metabolomics,rumen microbiology and meat quality (By Figdraw).

Finally, combining Figures 4C,B, we can observe the positive correlation between the crude fiber, ADF, and NDF contents in forages and the rumen microbe *Prevotellaceae_UCG-003.* Meanwhile, *Prevotellaceae_UCG-003* shows a significant positive correlation with N6 PUFA, C18:1 N9, and b* in the *longissimus dorsi* muscle of Tibetan sheep. In addition, crude protein, amino acids (such as proline, arginine, and aminocaproic acid), and amino acid metabolites (such as glutamine, proline, and caproic acid) in forages show significant positive correlations with the rumen microbes *F082* and *WCHB1-41*. Meanwhile, *F082* and *WCHB1-41* exhibit significant positive correlations with TAAs and cysteine in the *longissimus dorsi* muscle of Tibetan sheep. It is therefore (Figure 5) hypothesized that the up-regulated amino acid metabolism, high protein content, and low fiber content of the HG group forages altered the rumenal fermentation patterns in Tibetan sheep in the GC group. In response to the nutrient composition of the feed, the abundance of the rumen microbial community changed, with significantly increased abundance of *F082* and *WCHB1-41* associated with protein utilization and a significant decrease in the abundance of *Prevotellaceae_UCG-003* associated with fiber degradation. In turn, these alterations in rumen microbes affect the changes in amino acid metabolism and fatty acid contents of Tibetan sheep muscle, leading to the deposition of amino acids and reduced fatty acid contents, ultimately influencing the a* and b* values In addition, the large amounts of flavonoids accumulated in the pasture of the HG group exerted antioxidant functions, which could also influence changes in the muscle a* and b* values of Tibetan sheep.

## 5 Conclusion

Saline forages affects meat quality of Tibetan sheep. Specifically, Tibetan sheep in the GC group grazing on saline forage showed better meat color and significant increases in the total amino acid content resulting from the up-regulation of several amino acid metabolic pathways, including those of cysteine and methionine metabolism, as well as the accumulation of antioxidant components, such as flavonoids. In addition, saline pasture reduced the MUFA content in muscle by altering the rumen fermentation pattern of Tibetan sheep, resulting in significantly reduced abundance of the microorganism *Prevotellaceae_UCG-003*, which is associated with fiber degradation and fatty acid synthesis. In conclusion, the high protein and amino acid content and low levels of fiber (crude fiber, ADF, NDF) in saline grasses had a significant effect on maintaining muscle color, increasing the amino acid content, and reducing MUFA levels in Tibetan sheep.

## Data Availability

The original contributions presented in the study are publicly available. This data can be found here: https://www.ebi.ac.uk/metabolights/MTBLS12748.
