# Supplementary material for: Saline pasture improve meat quality in Qinghai Tibetan sheep through changes in the rumen microbiota
Source: Front Microbiol. 2025 Aug 5;16:1573040. doi: 10.3389/fmicb.2025.1573040 (PMC12365933; doi:10.3389/fmicb.2025.1573040)
Supplement: Supplementary file 1 [file Supplementary_file_1.docx]

1. **Supplementary Figures and Tables**

**1.1 Supplementary Figures**

**Supplementary Figures 1** Quality control of forage samples. A. The total ion chromatograms of quality control samples in positive ion modes. B. The total ion chromatograms of quality control samples in negative ion modes. C. Alignment test of PLS-DA in negative ion mode. D. Alignment test of OPLS-DA in negative ion mode. E. Volcano plots for comparison between HH and HG groups of forages in negative ion detection mode

**Supplementary Figures 2** Plot of PCA scores for all samples in positive (A) and negative (B) ion modes; PLS-DA (C) OPLS-DA (D) scores for overall samples in negative ion mode

**Supplementary Figures 3** Radar plot of sensory evaluation of Tibetan sheep in GC and HC groups

**Supplementary Figures 4** Quality control of meat samples. A. The total ion chromatograms of quality control samples in positive ion modes. B. The total ion chromatograms of quality control samples in negative ion modes. C. Alignment test of PLS-DA in positive ion mode. D. Alignment test of OPLS-DA in positive ion mode. E. Volcano plot for comparison between HC and GC groups in positive ion detection mode.


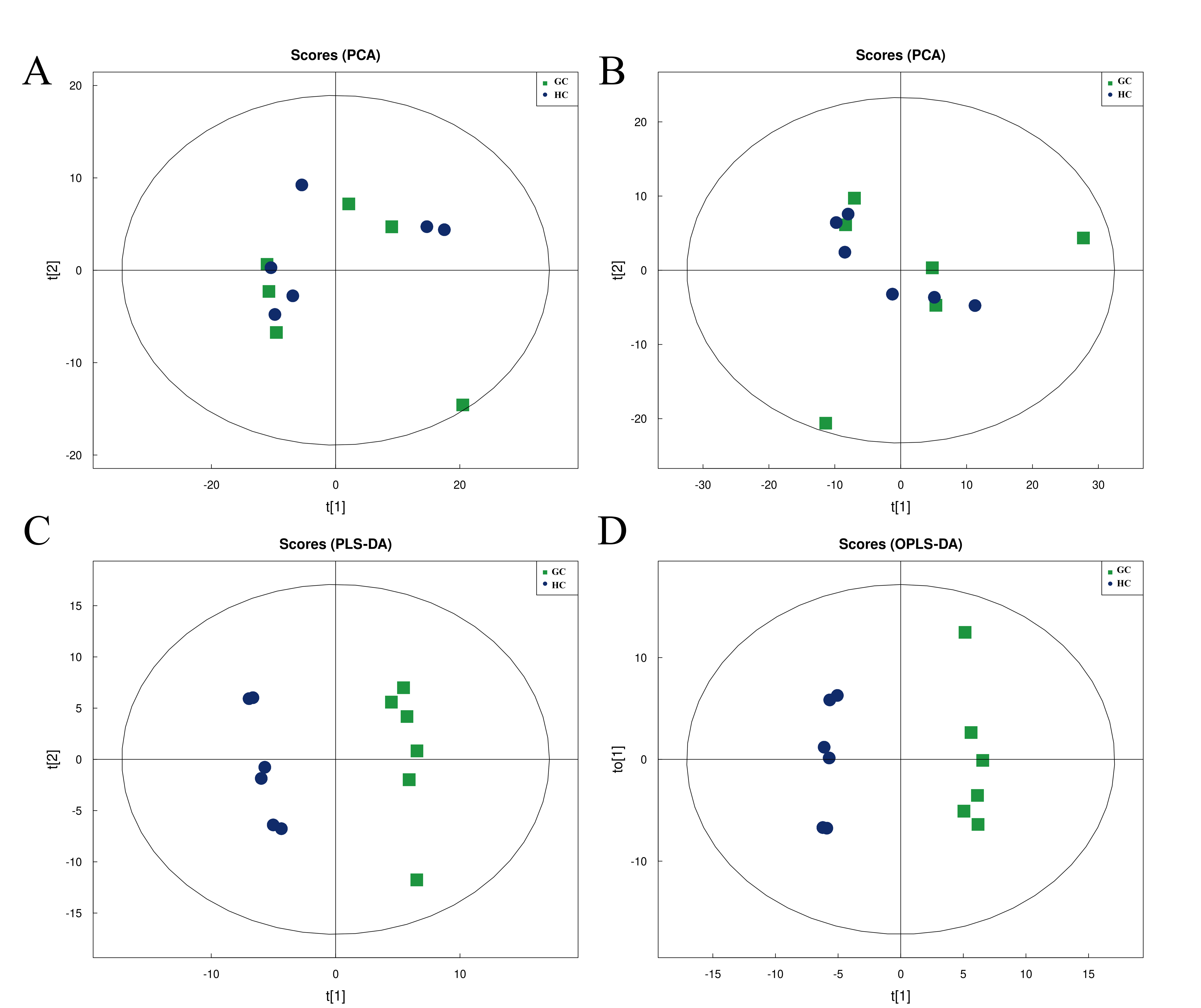


**Supplementary Figures 5** Plot of PCA scores for all samples in positive (A) and negative (B) ion modes; PLS-DA (C) OPLS-DA (D) scores for overall samples in positive ion mode

**1.2 Supplementary Tables**

**Supplementary Table 1**. Sensory scoring criteria for meat samples

| Evaluation project | Grading Standards | | | Assessment |
| --- | --- | --- | --- | --- |
|  | 1-4 | 5-7 | 8-10 |  |
| Appearance (Color) | The muscle color is yellowish or dark brown, and the fat is yellowish | The muscle is grayish, and the fat is white. | The muscle is light pink to grayish-white with a certain degree of luster, and the fat is white. |  |
| Fragrance | Lacking the meaty flavor. | The meaty flavor is relatively distinct. | The mutton flavor is rich and intense. |  |
| Muttony odor | The characteristic unpleasant odor of mutton is very noticeable. | The unpleasant odor is not pronounced. | The distinctive unpleasant odor of mutton is virtually absent. |  |
| Taste | The meat has a pronounced woody texture, is difficult to bite through, and has poor chewability. | The meat is slightly dry and astringent, with average chewability. | The meat is tender and palatable, with good chewability. |  |
| Texture | The tissue condition is poor, and the cooked meat appears in a fragmented state. | The tissue condition is average, with moderate elasticity. | The tissue condition is uniform and compact, with distinct fibers. |  |
| General acceptability | Not readily acceptable. | More readily acceptable. | Easily acceptable. |  |

**Supplementary Table 2**. Differential metabolites in pasture grasses in negative ion detection mode

| **Name** | **adduct** | **m/z** | **rt(s)** | **VIP** | **FC** | **Variation** | ***P*** |
| --- | --- | --- | --- | --- | --- | --- | --- |
| Carminic acid | [M-H-CO2]- | 447.09 | 484.86 | 2.35 | 3.52 | ↑ | <0.01 |
| (10e,15z)-9,12,13-trihydroxyoctadeca-10,15-dienoic acid | [M-H]- | 327.22 | 166.37 | 11.99 | 4.23 | ↑ | <0.01 |
| 1-palmitoyl-2-hydroxy-sn-glycero-3-phospho-(1'-rac-glycerol) | [M-H]- | 483.27 | 43.01 | 1.54 | 4.96 | ↑ | <0.01 |
| 8-o-acetylharpagide | [M-H]- | 405.14 | 251.21 | 1.82 | 11.22 | ↑ | <0.01 |
| Deoxyguanosine | [M-H]- | 266.09 | 249.22 | 1.84 | 32.34 | ↑ | <0.01 |
| His-ser | [M-H]- | 241.08 | 105.49 | 5.58 | 34.72 | ↑ | <0.01 |
| Ile-Pro | [M-H]- | 227.07 | 119.88 | 2.60 | 43.82 | ↑ | <0.01 |
| Creatine | [M-H]- | 130.05 | 403.70 | 1.76 | 0.04 | ↓ | <0.01 |
| Proline | [M-H]- | 114.06 | 331.17 | 4.74 | 0.06 | ↓ | <0.01 |
| Tyr-Tyr | [M-H]- | 343.11 | 35.57 | 1.61 | 0.12 | ↓ | <0.01 |
| D-Proline | (M-H)- | 114.06 | 369.80 | 1.18 | 0.17 | ↓ | <0.01 |
| Blood group b trisaccharide | [M-H]- | 487.17 | 405.45 | 1.59 | 0.24 | ↓ | <0.01 |
| 2-keto-3-deoxyoctonic acid | [M-H]- | 237.06 | 398.19 | 1.20 | 2.43 | ↑ | <0.01 |
| Thiamine monophosphate | [M-H]- | 343.08 | 37.76 | 2.03 | 0.10 | ↓ | <0.01 |
| Neobavaisoflavone | [M-H]- | 321.10 | 37.10 | 1.14 | 0.10 | ↓ | <0.01 |
| 2'-hydroxy-3,4,4',5-tetramethoxychalcone | [M-H]- | 343.10 | 34.88 | 1.87 | 0.10 | ↓ | <0.01 |
| 4-(3,4-dihydroxyphenyl)-7-hydroxy-5-[3,4,5-trihydroxy-6-[(3,4,5-trihydroxyoxan-2-yl)oxymethyl]oxan-2-yl]oxychromen-2-one | [M-H]- | 579.13 | 353.07 | 1.52 | 0.30 | ↓ | <0.01 |
| 3,4-dihydroxyhydrocinnamic acid | [M-H]- | 181.01 | 351.56 | 1.29 | 0.06 | ↓ | <0.01 |
| Hydroquinidine | [M-H]- | 325.18 | 31.27 | 8.14 | 2.82 | ↑ | <0.01 |
| Aurantio-obtusin beta-d-glucoside | [M-H]- | 491.12 | 133.76 | 4.09 | 5.50 | ↑ | 0.01 |
| Cis,cis-muconic acid | [M-H]- | 141.02 | 350.64 | 7.60 | 0.01 | ↓ | 0.01 |
| Fa 18:2+2o | [M-H]- | 311.22 | 72.85 | 1.82 | 2.21 | ↑ | 0.01 |
| Ostruthin | [M-H]- | 297.15 | 31.96 | 2.61 | 2.29 | ↑ | 0.01 |
| Palmitic acid | [M-H]- | 255.23 | 49.97 | 6.67 | 3.62 | ↑ | 0.01 |
| Guanidinosuccinic acid | [M-H]- | 174.04 | 403.46 | 1.26 | 0.06 | ↓ | 0.01 |
| Glutamine | [M-H]- | 145.06 | 407.72 | 2.01 | 0.44 | ↓ | 0.01 |
| Galactonic acid | (M-H)- | 195.05 | 519.60 | 2.26 | 4.71 | ↑ | 0.01 |
| Pantothenate | [M-H]- | 218.10 | 292.36 | 1.68 | 1.94 | ↑ | 0.01 |
| L-threonate | [M-H]- | 135.03 | 385.91 | 2.09 | 3.08 | ↑ | 0.01 |
| (2r,3s,4s,5r,6r)-2-[[(2s,3r,4r)-3,4-dihydroxy-4-(hydroxymethyl)oxolan-2-yl]oxymethyl]-6-(2-phenylethoxy)oxane-3,4,5-triol | [M-H]- | 415.14 | 440.19 | 2.81 | 4.05 | ↑ | 0.01 |
| D-Sorbitol | [M-H]- | 181.07 | 316.57 | 6.42 | 10.23 | ↑ | 0.01 |
| Fostriecin | [M-H-H2O]- | 411.13 | 141.56 | 2.19 | 0.06 | ↓ | 0.01 |
| Skullcapflavone ii | [M-H]- | 373.09 | 37.75 | 3.00 | 0.06 | ↓ | 0.01 |
| (e)-5-[(1s,4ar,8ar)-2-formyl-5,5,8a-trimethyl-1,4,4a,6,7,8-hexahydronaphthalen-1-yl]-3-(acetyloxymethyl)pent-2-enoic acid | [M-H]- | 375.20 | 200.73 | 1.19 | 0.09 | ↓ | 0.02 |
| Agnuside | [M-H]- | 465.14 | 145.53 | 2.27 | 0.10 | ↓ | 0.02 |
| Thymol-beta-d-glucoside | [M-H]- | 311.17 | 31.89 | 4.18 | 2.07 | ↑ | 0.02 |
| Pi(16:1/13-hode) | [M-H]- | 847.49 | 122.40 | 2.10 | 3.38 | ↑ | 0.02 |
| 9-hydroperoxy-10e,12z,15z-octadecatrienoic acid | [M-H]- | 309.21 | 109.22 | 1.88 | 3.90 | ↑ | 0.02 |
| Deoxyloganic acid (not validated) | [M-H]- | 359.13 | 203.63 | 2.26 | 5.30 | ↑ | 0.02 |
| Onopordopicrin | [M-H]- | 347.17 | 286.65 | 1.05 | 5.34 | ↑ | 0.02 |
| 9-oxo-10e,12z,15z-octadecatrienoic acid | [M-H]- | 291.19 | 40.48 | 1.94 | 6.28 | ↑ | 0.02 |
| Maltitol | [M-H]- | 343.12 | 418.96 | 1.29 | 9.16 | ↑ | 0.02 |
| Succinate | [M-H]- | 117.02 | 411.07 | 1.57 | 0.41 | ↓ | 0.02 |
| Myo-inositol | [M-H]- | 179.06 | 423.62 | 1.07 | 2.68 | ↑ | 0.02 |
| Coproporphyrin i | [M-H-CO2]- | 609.26 | 201.10 | 1.09 | 7.20 | ↑ | 0.02 |
| Haploperoside c acetate | [M+CH3COOH-H]- | 811.23 | 139.66 | 1.40 | 13.44 | ↑ | 0.02 |
| Stearidonic Acid | (M-H)- | 275.20 | 66.06 | 1.95 | 7.91 | ↑ | 0.02 |
| Scutellarioside ii | [M-H]- | 507.13 | 55.56 | 1.06 | 3.29 | ↑ | 0.03 |
| Sciadopitysin | [M-H]- | 579.13 | 487.23 | 2.12 | 3.97 | ↑ | 0.03 |
| (2r,3s,4s,5r,6r)-6-[1,7-bis(4-hydroxyphenyl)heptan-3-yloxy]-5-[(2s,3r,4r)-3,4-dihydroxy-4-(hydroxymethyl)oxolan-2-yl]oxy-2-(hydroxymethyl)oxane-3,4-diol | [M-H]- | 593.26 | 189.14 | 1.08 | 5.67 | ↑ | 0.03 |
| Idarubicin | [M-H-C3H15O6]- | 349.06 | 113.47 | 10.43 | 0.03 | ↓ | 0.03 |
| Flavone base + 3o, 2meo, o-guaiacylglycerol-hex | [M-H]- | 687.19 | 186.62 | 1.08 | 2.87 | ↑ | 0.03 |
| 13:4+4o fatty acyl hexoside | [M-H]- | 415.16 | 208.51 | 1.75 | 11.88 | ↑ | 0.03 |
| Fa 18:2+4o | [M-H]- | 343.21 | 209.48 | 1.13 | 2.43 | ↑ | 0.04 |
| 3-hydroxymyristic acid | [M-H]- | 243.20 | 97.36 | 1.78 | 39.21 | ↑ | 0.04 |
| 3-dehydroquinic acid | [M-H-H2O]- | 171.03 | 311.28 | 1.61 | 8.36 | ↑ | 0.04 |
| Cynarin | [M-H-C16H16O8]- | 179.06 | 378.35 | 2.66 | 0.40 | ↓ | 0.04 |
| Propanoic acid, 3-[[[2-[(aminoiminomethyl)amino]-4-thiazolyl]methyl]thio]- | [M+Cl]- | 294.99 | 503.09 | 1.64 | 0.35 | ↓ | 0.04 |
| Malvidin-3-o-glucoside | [M-2H]- | 491.12 | 180.06 | 2.35 | 5.12 | ↑ | 0.04 |
| Syringaresinol | [M-H]- | 417.15 | 182.16 | 1.49 | 0.30 | ↓ | 0.04 |
| 18-carboxydinorleukotriene b4 | [M-H]- | 337.15 | 304.34 | 7.45 | 0.01 | ↓ | 0.05 |
| 17-phenoxytrinorprostaglandin f2.alpha. | [M-H-H2O]- | 385.18 | 225.14 | 1.73 | 51.08 | ↑ | 0.05 |
| Marticin | [M-H]- | 375.07 | 107.18 | 2.08 | 0.05 | ↓ | 0.05 |
| 5(S)-HETE | (M+K-2H)- | 357.19 | 200.09 | 4.33 | 0.06 | ↓ | 0.05 |

**Supplementary Table 3.** Differential metabolites in the *Longissimus dorsi* muscle of Tibetan sheep in positive ion detection mode

| **Name** | **adduct** | **m/z** | **rt(s)** | **VIP** | **FC** | **Variation** | ***P*** |
| --- | --- | --- | --- | --- | --- | --- | --- |
| Trigonelline | [M+H]+ | 138.05 | 305.69 | 1.25 | 0.43 | ↓ | <0.01 |
| Diphenylamine | [M+H]+ | 170.12 | 353.24 | 4.59 | 0.26 | ↓ | <0.01 |
| Clomazon | [M+H]+ | 240.10 | 228.49 | 1.71 | 0.38 | ↓ | <0.01 |
| (2r)-3-hydroxyisovaleroylcarnitine | [M+H]+ | 262.16 | 308.41 | 5.69 | 0.55 | ↓ | <0.01 |
| Trans-4-(aminomethyl)cyclohexanecarboxylic acid | [M+H]+ | 158.12 | 274.83 | 14.67 | 0.05 | ↓ | <0.01 |
| Acetyl-dl-leucine | [M+H]+ | 174.11 | 385.32 | 1.64 | 0.39 | ↓ | <0.01 |
| (r)-aminocarnitine | [M+H]+ | 161.14 | 398.28 | 9.93 | 1.54 | ↑ | <0.01 |
| 6-dimethylamino-4-ketohexanoic acid | [M+H]+ | 174.11 | 349.36 | 5.34 | 3.15 | ↑ | <0.01 |
| Trimethylamine n-oxide | [2M+H]+ | 151.14 | 338.26 | 5.64 | 3.92 | ↑ | <0.01 |
| 5-oxo-1-propyl-2-pyrrolidineacetic acid | [M+H]+ | 186.11 | 343.42 | 1.04 | 1.99 | ↑ | <0.01 |
| N-nitrosopyrrolidine | [M+H]+ | 101.06 | 398.25 | 3.28 | 1.48 | ↑ | <0.01 |
| Normorphine | [M+H-H2O]+ | 254.12 | 213.66 | 1.11 | 0.17 | ↓ | 0.01 |
| 1,2-diarachidonoyl-sn-glycero-3-phosphocholine | [M+H]+ | 830.56 | 42.16 | 1.64 | 1.51 | ↑ | 0.01 |
| 5-aminovaleric acid betaine | [M+H]+ | 160.14 | 400.55 | 26.49 | 1.67 | ↑ | 0.01 |
| Deoxyadenosine | [M+H-C5H8O3]+ | 136.06 | 109.34 | 3.33 | 0.56 | ↓ | 0.01 |
| L-homoserine | [M+H-H2O]+ | 102.05 | 55.25 | 1.12 | 0.46 | ↓ | 0.01 |
| Glutaraldehyde | [M+H-H2O]+ | 83.05 | 398.83 | 1.01 | 1.47 | ↑ | 0.01 |
| 3-hydroxybutyrylcarnitine | [M+H]+ | 248.15 | 334.89 | 14.33 | 0.36 | ↓ | 0.02 |
| S-methyl-5'-thioadenosine | [M+H]+ | 298.10 | 110.01 | 9.08 | 0.53 | ↓ | 0.02 |
| 1-Methylhistidine | (M+H)+ | 170.09 | 346.34 | 3.84 | 0.23 | ↓ | 0.02 |
| 4-hydroxy-l-isoleucine | [M+H-CH2O2]+ | 102.09 | 372.01 | 2.63 | 1.58 | ↑ | 0.02 |
| L-homocitrulline | [M+H]+ | 190.12 | 413.06 | 1.04 | 1.75 | ↑ | 0.02 |
| 2-oleoyl-1-stearoyl-sn-glycero-3-phosphoserine | [M+H]+ | 790.57 | 42.55 | 2.68 | 1.72 | ↑ | 0.03 |
| 1-palmitoyl-2-docosahexaenoyl-sn-glycero-3-phosphocholine | [M+H]+ | 806.56 | 42.57 | 3.02 | 1.72 | ↑ | 0.03 |
| Glycerophosphocholine | [M+H]+ | 258.11 | 400.58 | 18.23 | 0.62 | ↓ | 0.04 |
| Dl-threonine methyl ester | [M+H]+ | 134.08 | 74.26 | 2.87 | 0.50 | ↓ | 0.04 |
| Atrazine-desisopropyl-2-hydroxy | [M+H]+ | 156.10 | 414.38 | 1.46 | 0.33 | ↓ | 0.04 |
| Desipramine | [M+H]+ | 267.20 | 270.78 | 2.26 | 16.57 | ↑ | 0.04 |
| Choline | [M]+ | 104.11 | 400.81 | 2.44 | 0.68 | ↓ | 0.05 |

**Supplementary Table 4.** Changes in differential metabolites comparing key metabolic pathways in samples from the GC and HC cohorts (absolute differential abundance scores of ≥0.5 for all metabolic pathways)

| Metabolic pathways | Metabolites |
| --- | --- |
| Downregulation in the GC group HC VS GC | |
| Two-component system | Trimethylamine n-oxide |
| Upregulation in the GC group HC VS GC | |
| Glycine, serine and threonine metabolism | Choline |
|  | L-homoserine |
| Glycerophospholipid metabolism | Choline |
|  | Glycerophosphocholine |
| Cysteine and methionine metabolism | L-homoserine |
|  | S-methyl-5'-thioadenosine |
| Cholinergic synapse | Choline |
| Atrazine degradation | Atrazine-desisopropyl-2-hydroxy |
| Ether lipid metabolism | Glycerophosphocholine |
| ABC transporters | Choline |
|  | Deoxyadenosine |
| Sulfur metabolism | L-homoserine |
| Lysine biosynthesis | L-homoserine |
| Histidine metabolism | 1-Methylhistidine |

**Supplementary Table 5.** Effect of forage diets from different regions on the α diversity of rumen bacteria in Tibetan sheep

|  | GC | HC | *P*-value |
| --- | --- | --- | --- |
| ace | 1814.06±293.47 | 1669.73±281.62 | 0.41 |
| chao1 | 1827.72±308.48 | 1679.03±287.73 | 0.41 |
| shannon | 9.24±0.24 | 8.71±0.31 | 0.01 |
| simpson | 0.995±0.00 | 0.991±0.00 | <0.01 |

**Supplementary Table 6.** Regional Variations in Amino Acid and Fatty Acid Profiles of Natural Forage Grasses: A Complete Dataset

| parameters | HG | HH | *P*-value |
| --- | --- | --- | --- |
| AA (mg/100g) | | | |
| Alanine | 177.60±4.24 | 269.68±1.61** | <0.01 |
| Aminoadipic acid | 15.59±1.53** | 4.17±0.15 | 0.01 |
| Arginine | 1678.43±92.45** | 710.93±234.20 | 0.01 |
| Asparagine | 16.62±0.05 | 22.53±0.61** | <0.01 |
| Aspartate | 110.72±2.45 | 106.22±1.40 | 0.07 |
| Choline | 217.28±1.65 | 254.43±4.07** | <0.01 |
| Citrulline | 1.96±0.14** | 0.86±0.07 | <0.01 |
| Creatine | 0.37±0.01 | 0.62±0.01** | <0.01 |
| Creatinine | 0.05±0.00** | 0.01±0.00 | <0.01 |
| Cysteine | 0.07±0.01* | 0.06±0.00 | 0.04 |
| Cystine | 0.84±0.00** | 0.44±0.00 | <0.01 |
| Glutamate | 811.07±4.52** | 668.89±6.32 | <0.01 |
| Glutamine | 0.02±0.01 | 0.07±0.01** | <0.01 |
| Glycine | 509.08±59.50* | 275.31±8.66 | 0.02 |
| Histidine | 2194.89±90.68** | 963.16±266.49 | 0.01 |
| Hydroxyproline | 1.45±0.00** | 0.55±0.02 | <0.01 |
| Isoleucine | 361.49±6.88 | 425.51±5.33** | <0.01 |
| Leucine | 320.35±20.37 | 358.79±12.56 | 0.06 |
| Lysine | 7.55±0.16 | 11.32±2.60 | 0.13 |
| Methionine | 29.71±0.35 | 28.95±0.40 | 0.07 |
| Ornithine | 0.27±0.02 | 0.46±0.02** | <0.01 |
| Phenylalanine | 965.98±38.67 | 1077.94±0.77* | 0.04 |
| Proline | 4740.09±228.26** | 554.81±10.61 | <0.01 |
| Serine | 1202.01±19.60 | 1406.31±7.02** | <0.01 |
| Spermidine | 1.27±0.19 | 1.92±0.77 | 0.27 |
| Taurine | 0.04±0.01 | 0.42±0.05** | <0.01 |
| Threonine | 1637.87±18.88 | 3171.25±27.81** | <0.01 |
| Tryptophan | 7.93±0.41 | 7.84±0.20 | 0.78 |
| Tyrosine | 232.26±12.58 | 236.86±4.35 | 0.60 |
| Valine | 1531.05±29.34** | 1025.76±3.41 | <0.01 |
| EAAs | 4861.93±22.14 | 6107.38±19.95** | <0.01 |
| NEAAs | 11912.38±430.30** | 5479.39±485.33 | <0.01 |
| TAAs | 16774.31±452.44** | 11586.76±465.38 | <0.01 |
| FA (mg/100g) | | | |
| c6:0 | 0.01±0.00 | 0.01±0.00 | 0.90 |
| c8:0 | 0.06±0.02 | 0.07±0.00 | 0.44 |
| c10:0 | 0.35±0.09* | 0.12±0.01 | 0.05 |
| c11:0 | 0.01±0.00 | 0.02±0.00** | 0.01 |
| c12:0 | 1.40±0.23 | 1.92±0.02 | 0.06 |
| c13:0 | 0.049±0.00 | 0.051±0.00 | 0.17 |
| c14:0 | 3.62±0.08 | 3.67±0.01 | 0.41 |
| c14:1n5 | 0.14±0.00 | 0.18±0.01 | 0.06 |
| c15:0 | 0.44±0.01 | 0.46±0.00* | 0.02 |
| c15:1n5 | 2.35±0.15 | 2.20±0.09 | 0.24 |
| c16:0 | 49.81±0.27 | 52.57±0.63** | 0.01 |
| c16:1n7 | 0.71±0.01 | 1.02±0.01** | <0.01 |
| c17:0 | 1.142±0.00 | 1.141±0.00 | 0.83 |
| c17:1n7 | 0.19±0.04 | 0.38±0.03** | <0.01 |
| c18:0 | 10.78±0.05** | 8.79±0.20 | <0.01 |
| c18:1n9 | 36.53±2.46** | 13.30±0.45 | <0.01 |
| c18:2n6 | 82.88±3.61** | 53.30±0.25 | 0.01 |
| c18:3n6 | 0.01±0.00 | 0.03±0.00** | 0.01 |
| c18:3n3 | 110.57±2.39 | 141.96±0.74** | <0.01 |
| c20:0 | 3.59±0.31 | 3.39±0.01 | 0.38 |
| c20:1n9 | 2.22±0.09 | 2.48±0.08* | 0.02 |
| c20:2n6 | 0.22±0.00** | 0.20±0.00 | <0.01 |
| c20:3n3 | 0.24±0.03 | 0.39±0.02** | <0.01 |
| c20:5n3 | 2.26±0.07 | 2.20±0.01 | 0.32 |
| c21:0 | 0.40±0.01 | 0.39±0.00 | 0.30 |
| c22:0 | 0.04±0.00 | 0.05±0.00* | 0.05 |
| c22:2n6 | 0.06±0.00* | 0.05±0.00 | 0.02 |
| c22:5n3 | 0.10±0.00 | 0.12±0.00** | <0.01 |
| c22:5n6 | 0.06±0.00** | 0.04±0.00 | <0.01 |
| c22:6n3 | 0.14±0.00 | 0.90±0.01 | 0.16 |
| c23:0 | 0.90±0.01* | 0.86±0.01 | 0.02 |
| c24:0 | 4.14±0.15** | 2.58±0.02 | <0.01 |
| c24:1n9 | 0.37±0.05 | 0.78±0.05** | <0.01 |
| SFA | 76.72±1.11 | 76.09±0.46 | 0.44 |
| MUFA | 42.51±2.74** | 20.35±0.44 | <0.01 |
| PUFA | 196.53±1.28 | 198.43±0.98 | 0.12 |
| N6 | 83.23±3.61** | 53.62±0.25 | 0.01 |
| N3 | 113.31±2.33 | 144.81±0.73** | <0.01 |
| N6/N3 | 0.74±0.05* | 0.37±0.00 | 0.01 |
| PUFA/SFA | 2.56±0.02 | 2.61±0.00 | 0.06 |

Note: Values (mean ± SD). * *P* < 0.05 and ** *P* < 0.01.

**Supplementary Table 7.** Analysis of amino acid and fatty acid composition and content in the longest dorsal muscle of Tibetan sheep: A Complete Dataset

| parameters | GC | HC | *P*-value |
| --- | --- | --- | --- |
| AA (mg/100g) | | | |
| Alanine | 127.27±6.08 | 115.26±2.65 | 0.06 |
| Aminoadipic acid | 1.18±0.07* | 1.03±0.06 | 0.05 |
| Arginine | 1091.84±50.57* | 969.69±51.88 | 0.04 |
| Asparagine | 0.61±0.02* | 0.56±0.02 | 0.05 |
| Aspartate | 144.67±29.57* | 70.57±2.07 | 0.05 |
| Choline | 67.67±2.52 | 63.77±4.11 | 0.25 |
| Citrulline | 12.50±0.01** | 7.22±0.10 | <0.01 |
| Creatine | 173.79±2.38* | 163.33±3.38 | 0.02 |
| Creatinine | 1.37±0.00 | 1.53±0.01** | <0.01 |
| Cysteine | 0.05±0.00** | 0.03±0.00 | <0.01 |
| Cystine | 41.09±1.92 | 54.72±1.00** | <0.01 |
| Glutamate | 34.04±0.97 | 55.29±2.54** | <0.01 |
| Glutamine | 18.50±0.66** | 13.99±0.64 | <0.01 |
| Glycine | 737.31±3.11** | 554.31±6.88 | <0.01 |
| Histidine | 297.82±8.06 | 304.11±7.25 | 0.37 |
| Hydroxyproline | 0.58±0.03 | 0.68±0.02** | 0.01 |
| Isoleucine | 191.71±8.06 | 175.83±0.71 | 0.08 |
| Leucine | 384.72±10.90 | 369.20±4.64 | 0.12 |
| Lysine | 316.94±28.92 | 329.22±15.42 | 0.56 |
| Methionine | 32.97±2.48 | 28.05±0.28 | 0.07 |
| Ornithine | 0.37±0.02 | 0.40±0.02 | 0.21 |
| Phenylalanine | 246.15±13.11 | 248.15±12.92 | 0.86 |
| Proline | 177.21±7.45* | 156.80±7.03 | 0.03 |
| Serine | 228.62±13.64* | 183.57±4.11 | 0.02 |
| Spermidine | 0.020±0.00** | 0.018±0.00 | <0.01 |
| Taurine | 12.47±1.17 | 20.16±2.83* | 0.03 |
| Threonine | 227.97±4.28 | 228.05±3.54 | 0.98 |
| Tryptophan | 1.84±0.10* | 1.62±0.03 | 0.05 |
| Tyrosine | 232.48±18.64 | 194.32±5.98 | 0.06 |
| Valine | 354.29±18.43 | 360.20±4.24 | 0.64 |
| EAAs | 1756.58±81.12 | 1740.31±7.40 | 0.76 |
| NEAAs | 3401.47±68.37** | 2931.35±50.84 | <0.01 |
| TAAs | 5158.05±149.48* | 4671.66±58.24 | 0.02 |
| FA (mg/100g) | | | |
| C4:0 | 0.16±0.03 | 0.16±0.00 | 0.85 |
| C6:0 | 0.10±0.01** | 0.07±0.01 | 0.01 |
| C8:0 | 0.065±0.01 | 0.066±0.01 | 0.87 |
| C10:0 | 0.40±0.00 | 0.26±0.06 | 0.06 |
| C11:0 | 0.009±0.00 | 0.011±0.00 | 0.16 |
| C12:0 | 0.29±0.01 | 0.25±0.05 | 0.29 |
| C13:0 | 0.02±0.00 | 0.01±0.00 | 0.06 |
| C14:0 | 1.53±0.12 | 1.94±0.31 | 0.14 |
| C14:1N5 | 0.06±0.00 | 0.09±0.01** | 0.01 |
| C15:0 | 0.78±0.09 | 0.74±0.10 | 0.64 |
| C15:1N5 | 0.04±0.00 | 0.05±0.00* | 0.02 |
| C16:0 | 15.53±1.34 | 14.70±1.00 | 0.44 |
| C16:1N7 | 0.87±0.02 | 1.43±0.05** | <0.01 |
| C17:0 | 1.19±0.07 | 1.83±0.13** | <0.01 |
| C17:1N7 | 1.15±0.13 | 1.69±0.07** | 0.01 |
| C18:0 | 11.63±0.44 | 13.20±0.87 | 0.07 |
| C18:1TN9 | 50.01±1.91 | 56.77±3.72 | 0.07 |
| C18:1N9 | 19.60±1.65 | 26.29±1.70** | 0.01 |
| C18:2TTN6 | 0.34±0.01* | 0.20±0.03 | 0.02 |
| C18:2N6 | 12.48±0.43 | 15.67±1.67 | 0.07 |
| C18:3N6 | 0.67±0.03 | 0.88±0.02** | <0.01 |
| C18:3N3 | 12.77±1.09 | 11.34±0.70 | 0.14 |
| C20:0 | 0.09±0.00 | 0.10±0.01 | 0.40 |
| C20:1N9 | 0.10±0.00 | 0.13±0.02 | 0.19 |
| C20:2N6 | 1.20±0.16 | 1.16±0.13 | 0.78 |
| C20:3N6 | 1.60±0.01* | 1.26±0.13 | 0.05 |
| C20:4N6 | 7.72±0.16 | 10.50±0.91* | 0.03 |
| C20:5N3 | 7.34±0.11** | 3.61±0.47 | <0.01 |
| C21:0 | 0.18±0.00 | 0.20±0.01 | 0.07 |
| C22:0 | 0.09±0.01 | 0.14±0.01** | 0.01 |
| C22:4N6 | 0.27±0.00 | 0.46±0.03** | 0.01 |
| C22:5N3 | 5.45±0.15* | 3.65±0.60 | 0.03 |
| C22:5N6 | 0.06±0.00 | 0.08±0.00* | 0.02 |
| C22:6N3 | 1.34±0.11** | 0.91±0.10 | 0.01 |
| C23:0 | 0.81±0.03 | 0.86±0.04 | 0.20 |
| C24:0 | 0.30±0.04 | 0.48±0.03** | 0.01 |
| C24:1N9 | 2.01±0.14 | 2.39±0.29 | 0.13 |
| SFA | 33.17±2.20 | 35.02±2.37 | 0.38 |
| MUFA | 73.84±3.85 | 88.84±5.85* | 0.03 |
| PUFA | 51.35±1.83 | 49.86±1.85 | 0.38 |
| N6 | 24.34±0.38 | 30.23±2.30* | 0.04 |
| N3 | 26.90±1.46** | 19.50±0.47 | 0.01 |
| N6/N3 | 0.91±0.04 | 1.55±0.16* | 0.02 |
| PUFA/SFA | 1.55±0.05* | 1.43±0.04 | 0.03 |

Note: Values (mean ± SD). * *P* < 0.05 and ** *P* < 0.01.
